# Machine Learning and Probabilistic Approaches for Forecasting COVID-19 Transmission and Cases

**DOI:** 10.1101/2025.06.24.25330210

**Published:** 2025-06-24

**Authors:** Md Sakhawat Hossain, Ravi Goyal, Natasha K Martin, Victor DeGruttola, Tanvir Ahammed, Christopher McMahan, Lior Rennert

**Affiliations:** 1 Department of Public Health Sciences, Clemson University, Clemson, SC, USA; 2 Center for Public Health Modeling and Response, Clemson University, Clemson, SC, USA; 3 Division of Infectious Diseases & Global Public Health, University of California San Diego, La Jolla, CA, USA; 4 Division of Biostatistics, Herbert Wertheim School of Public Health and Longevity Science, University of California San Diego, San Diego, California, USA; 5 School of Mathematical and Statistical Sciences, Clemson University, Clemson, SC, USA

**Keywords:** Effective reproductive number, Infectious disease modeling, Machine learning, Forecasting, COVID-19

## Abstract

Forecasting the effective reproductive number ($R_{t}$) and COVID-19 case counts are critical for guiding public health responses. We developed a machine learning and probabilistic forecasting framework to predict $R_{t}$ and daily case counts at the county level in South Carolina (SC). Our approach utilized initial $R_{t}$ estimates from EpiNow2 R package refined with spatial (covariate-adjusted) smoothing. We then generated $R_{t}$ forecasts using an ensemble of regression, Random Forest, and XGBoost models, and predicted case counts with a probabilistic Poisson model.

This ensemble-based approach consistently outperformed EpiNow2 across different forecast horizons (7-day, 14-day, and 21-day). In the first forecast period (November 11, 2020 – February 02, 2021), the ensemble achieved a median percentage agreement (PA) across counties of 94.4% (IQR: 93.8% – 95.3%) for 7-day ahead $R_{t}$ forecast, compared to 87.0% (IQR: 84.4% – 89.4%) from EpiNow2. In the second period (December 11, 2022 – March 04, 2023), the ensemble attained a 93.0% median PA across counties for Rt forecast (IQR: 91.3% – 94.1%), while EpiNow2 reached 86.8% (IQR: 82.5% – 89.2%). Similar trends were observed for case forecast, with the ensemble model demonstrating improved stability and performance. Combining spatial smoothing with ensemble modeling improves epidemic forecasting by enhancing predictive performance and robustness.

## 1 Introduction

The COVID-19 pandemic has significantly reshaped global priorities in public health, focusing on the importance of data-driven models to predict and manage disease spread. A crucial parameter in understanding and forecasting infectious diseases is the effective reproductive number ($R_{t}$), which indicates the average number of secondary cases caused by an infected individual at a given time t. In contrast to the basic reproduction number ($R_{0}$), which assumes a completely susceptible population, $R_{t}$ changes over time during an epidemic due to factors such as the reduction of susceptible population, changes in contact behavior, seasonal pathogen variations, and interventions (pharmaceutical and non-pharmaceutical) (Ali et al., 2020; Anderson and May, 1992; Delamater et al., 2019; Flaxman et al., 2020; Nishiura et al., 2020a; Nishiura and Chowell, 2009). This parameter is widely utilized to monitor and assess outbreak progression and has played a critical role in informing public health interventions and policy decisions during the COVID-19 pandemic (Gostic et al., 2020; Huisman et al., 2022). Values greater than 1 indicate disease transmission is increasing, while values below 1 indicate the disease is contracting (Anderson and May, 1992; Nishiura and Chowell, 2009).

Accurately estimating and forecasting $R_{t}$ is crucial for assessing the transmissibility of infectious disease and guiding public health interventions, including social distancing, mask mandates, vaccination strategies, and resource allocation (Cori et al., 2013; Flaxman et al., 2020; Fraser, 2007; Gostic et al., 2020; Kucharski et al., 2020). The $R_{t}$ estimates can be linked to changes in policy, population behavior and immunity, pathogen evolution, and other factors (Banholzer et al., 2022; Gostic et al., 2020; Kucharski et al., 2020; Lison et al., 2022). Moreover, $R_{t}$ is a critical input in forecasting models that estimate the daily infections, hospital admissions and resource utilization; such estimates aid in developing informed public health decisions and effective interventions (Nash et al., 2022).

Various methods have been developed to estimate $R_{t}$, including those capable of real-time monitoring, each with its unique strengths and limitations (Abbott et al., 2020; Bettencourt and Ribeiro, 2008; Cauchemez et al., 2006; Cori et al., 2013; Gostic et al., 2020; Meyer et al., 2017; Parag, 2021; Thompson et al., 2019; Wallinga and Lipsitch, 2006; Wallinga and Teunis, 2004). EpiNow2 enhanced estimation accuracy by incorporating generation time intervals, incubation period, and reporting delays (Abbott et al., 2020; Gostic et al., 2020), and EpiFilter employs recursive filtering to refine estimates, particularly in low incidence settings (Parag, 2021). Most methods focus on retrospective estimation of $R_{t}$ rather than future prediction. Overall, these estimation methodologies focus on interpreting past transmission trends, but not a forward-looking prediction. There is no standardized framework or widely adopted tool for forecasting $R_{t}$. Moreover, while epidemic forecasting is growing field, much of it focused on predicting case counts and other metrics, with relatively fewer studies explicitly forecasting the future course of $R_{t}$. Integrating machine learning techniques can improve forecasting accuracy, enable real-time adjustments, and enhance epidemic prediction tools for more effective public health decision-making (Ardabili et al., 2020; Chakraborty and Ghosh, 2020; Petropoulos and Makridakis, 2020; Shinde et al., 2020).

Machine learning has significantly advanced infectious disease modeling, aiding in outbreak detection, diagnosis, severity prediction, and forecasting of $R_{t}$ and case counts (Arik et al., 2020; Gao et al., 2020; Mei et al., 2020; Wynants et al., 2020). Supervised learning techniques, such as Random Forest (RF) and gradient boosting, have been widely used for predicting disease incidence and hospitalizations, often outperforming traditional regression methods (Fife and D’Onofrio, 2023; King and Strumpf, 2022). Neural networks, including recurrent neural networks (RNNs) and long short-term memory (LSTM) models, capture temporal dependencies, while artificial neural networks (ANNs) incorporate epidemiological indicators for $R_{t}$ prediction, though their accuracy depends on large datasets and hyperparameter tuning (Gatto et al., 2022). Despite RF’s strong performance, some studies suggest XGBoost (XGB) outperforms RF in optimized settings (Muehlensiepen et al., 2024; Song et al., 2022). Combining forecasts from multiple models has emerged as a powerful approach for epidemic forecasting. Ensemble methods that combine outputs from multiple models have demonstrated improved robustness (Oidtman et al., 2021), particularly in scenarios with inconsistent or incomplete data (Arik et al., 2020).

In this study, we develop a framework for forecasting the effective reproductive number ($R_{t}$) and, subsequently, daily case counts. First, we estimate $R_{t}$ using the R *software package* EpiNow2. Next, we apply a spatial (covariate-adjusted) smoothing through INLA method to refine the $R_{t}$ estimates (Hossain et al., 2024). We then employ regression and machine learning models, such as RF and XGB, to forecast $R_{t}$ using both the initial and smooth estimates. Finally, a stochastic Poisson process is implemented to generate COVID-19 case counts based on $R_{t}$ forecasts, utilizing historical data and the disease serial interval distribution.

## 2 Materials and Methods

In this study, we develop a forecasting framework to predict the effective reproductive number ($R_{t}$) and daily COVID-19 case counts. We apply our method to predict these quantities for 46 South Carolina (SC) counties. The methodology integrates machine learning techniques with probabilistic modeling to improve forecasting accuracy. First, $R_{t}$ is estimated using the EpiNow2 R package, which applies Bayesian time-series modeling, accounting for reporting delays and incubation periods. These estimates are then refined using a spatial (covariate-adjusted) smoothing process through the Integrated Nested Laplace Approximation (INLA) method.

To forecast $R_{t}$, we employ Regression (Reg), XGBoost (XGB), and Random Forest (RF) models, leveraging lagged values of the $R_{t}$ estimates and temporal features such as the day of the year. A recursive approach is applied to generate multi-step-ahead forecasts, iteratively updating predictions based on prior estimates. An ensemble-based forecasting approach is then developed by combining outputs from individual models.

**Figure 1.**
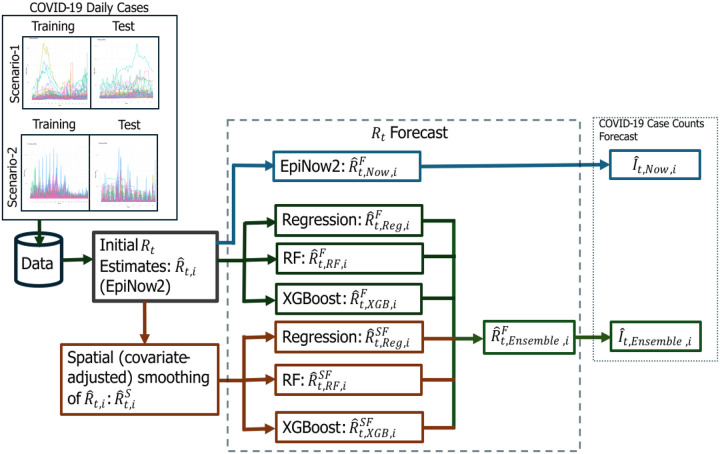
Flow diagram of the forecasting framework for effective reproductive number ($R_{t}$) and COVID-19 case counts at the county level in South Carolina (SC). Scenario-1 includes the data from June 01, 2020, to November 10, 2020 (Training) and November 11, 2020, to February 02, 2021 (Test, forecast period), while Scenario-2 covers data from May 01, 2022, to December 10, 2022 (Training) and December 11, 2022, to March 04, 2023 (Test, forecast period). Initially, $R_{t}$ is estimated using the EpiNow2 package, followed by spatial (covariate-adjusted) smoothing via the INLA model. The initial and smoothed estimates are denoted as ${\hat{R}}_{t,i}$ and ${\hat{R}}_{t,i}^{S}$, respectively. $R_{t}$ forecasting is performed using Regression (Reg), Random Forest (RF), and XGBoost (XGB) on both initial and spatially smoothed estimates, followed by an ensemble-based approach for final predictions. The ensemble forecasted $R_{t}$ is denoted as ${\hat{R}}_{t,\text{Ensemble},i}^{F}$, while ${\hat{I}}_{t,\text{Ensemble},i}$ and ${\hat{I}}_{t,\text{Now},i}$ represent COVID-19 case count forecasts from the ensemble approach and EpiNow2, respectively.

For COVID-19 case forecasting, we use a stochastic Poisson process, linking the forecasted $R_{t}$ values with historical case data and the serial interval distribution to estimate the expected number of daily infections. Forecasts are generated recursively, incorporating updated case counts at each step to refine predictions. The final forecasting performance is evaluated using percentage agreement (PA) to assess accuracy across different forecast horizons. Figure 1 presents the workflow diagram of $R_{t}$ and daily COVID-19 case forecasts.

We perform the initial estimation of $R_{t}$ for the period June 01, 2020, to March 04, 2023. The forecasts for both $R_{t}$ and COVID-19 daily case counts are generated for the time periods November 11, 2020 – February 02, 2021 (Scenario-1) and December 11, 2022 – March 04, 2023 (Scenario-2). The forecasting approach involves 7-day, 14-day, and 21-day ahead predictions over 84 days period using a rolling window method, ensuring that new data is continuously incorporated into the forecasting models to refine predictions. We employ Reg, RF, and XGB models on both the initial estimates and spatially smoothed estimates for forecasting $R_{t}$. All models are trained on $R_{t}$ estimates from June 01, 2020, to November 10, 2020, for forecasting under Scenario-1, and from May 01, 2022, to December 10, 2022, for Scenario-2. Then we generate $R_{t}$ forecasts through an ensemble-based approach that combines predictions from all individual models. To benchmark the performance of our models, we also produce $R_{t}$ and case forecasts using the EpiNow2 R package, however, these forecasts are used only for comparison purposes and are not included in the ensemble.

### 2.1 Data sources

We utilize publicly available county-level COVID-19 daily case data for South Carolina (SC) from the New York Times (NYT) GitHub repository (The New York Times, 2021), which provides daily case counts at the county level. For this analysis, we use data spanning from June 01, 2020, to March 04, 2023. The demographic variables, including population by age, sex, race/ethnicity, employment, and insurance coverage are used. These data are sourced from the United States Census Bureau website (U.S. Census Bureau, 2020). The social vulnerability index (SVI) data is obtained from the Agency for Toxic Substances and Disease Registry website (Agency for Toxic Substances and Disease Registry, 2022).

### 2.2 Initial estimation and spatial (covariate-adjusted) smoothing of $R_{t}$

We first utilize the EpiNow2 R package to estimate the effective reproductive number, $R_{t}$. It estimated time-varying $R_{t}$ from the cases at a given time t. It incorporates cases by reported date, generation time interval, delay distributions such as incubation and reporting delays, more details can be found at Abbott et al. (Abbott et al., 2020). The incubation period distribution and the reporting delay distribution are typically modeled using gamma or log-normal distributions, in this study, we used a gamma distribution. For our analysis, we consider a mean incubation period of 5 days (95% CI, 4.94–5.06) with a standard deviation of 2.4 days (Grant et al., 2022), a mean serial interval distribution of 4.7 days with a standard deviation of 2.9 days (Nishiura et al., 2020b), and a mean reporting delays of 3.2 days (Harris, 2022). The initial estimate of $R_{t}$ using EpiNow2 at a given time t and for a specific region i is denoted by ${\hat{R}}_{t,i}$. To spatially smooth $R_{t}$ estimates, a spatial (covariate-adjusted) Bayesian model is employed, as described in (Hossain et al., 2024). The model incorporates sociodemographic covariates, including age, race, employment, insurance status, Social Vulnerability Index (SVI), and normalized median income. We denote the smoothed estimates by ${\hat{R}}_{t,i}^{S}$.

### 2.3 Forecasting effective reproductive number ($R_{t}$)

In this section, we outline the methodology for forecasting the effective reproductive number utilizing both estimates ${\hat{R}}_{t,i}$ and ${\hat{R}}_{t,i}^{S}$ across M regions. These forecasts are denoted as ${\hat{R}}_{t,i}^{F}$ and ${\hat{R}}_{t,i}^{\text{SF}}$ for region i at time t. ${\hat{R}}_{t,i}$ and ${\hat{R}}_{t,i}^{S}$ are treated as time series data. For generalization, we represent these time series as $y_{t,i}$, corresponding to either ${\hat{R}}_{t,i}$ or ${\hat{R}}_{t,i}^{S}$. We consider the lagged estimates as predictors and incorporate temporal features, such as the day of the year (D). Recursive forecasting is performed to predict the future values for h-steps ahead using three methods: regression, RF, and XGB models. The RF algorithm, a widely used ensemble learning method, builds multiple decision trees during training and outputs the average prediction from all trees (Breiman, 2001). Its ability to handle non-linearity and interactions between features makes it suitable for $R_{t}$ forecasting. XGBoost, an optimized implementation of gradient boosting, builds multiple predictive models by sequentially adding decision trees to minimize the prediction error (Chen and Guestrin, 2016). It is known for its efficiency, scalability, and strong performance, particularly in time series and forecasting tasks.

The time series data for region i is $y_{1,i},y_{2,i},\ldots ,y_{T,i}$, where T is the most recent date used for forecasting. The goal is to forecast $y_{T+h,i}$ for h∈{1,2,…,H}, where H is the forecasting horizon. For each region i, the features are constructed using p lagged values of the time series and the day of the year corresponding to the forecasted time point ($D_{t+h}$).

The feature matrix for all regions is constructed as below:

$$X=\left(\begin{array}{ccccc} y_{1,1} & y_{2,1} & \cdots & y_{p,1} & D_{p+1,1}\\ y_{2,1} & y_{3,1} & \cdots & y_{p+1,1} & D_{p+2,1}\\ \vdots & \vdots & \ddots & \vdots & \vdots \\ y_{T-p,1} & y_{T-p+1,1} & \cdots & y_{T-1,1} & D_{T,1}\\ y_{1,2} & y_{2,2} & \cdots & y_{p,2} & D_{p+1,2}\\ y_{2,2} & y_{3,2} & \cdots & y_{p+1,2} & D_{p+2,2}\\ \vdots & \vdots & \ddots & \vdots & \vdots \\ y_{T-p,M} & y_{T-p+1,M} & \cdots & y_{T-1,M} & D_{T,M} \end{array}\right)$$

The response vector is denoted as:

$$y={\left(y_{p+1,1}\;y_{p+2},1\;\cdots \;y_{T,1}\;y_{p+1,2}\;y_{p+2,2}\;\cdots \;y_{T,M}\right)}^{\prime }.$$

Then the model is expressed as:

y=Xβ+ϵ

where y is response vector of (T−p). M elements, X is features matrix of (T−p). M rows and (p+1) columns, β is the column vector of model parameters learned during the training, and ϵ is the residual error term.

To determine the optimal number of lagged features ($p^{*}$) for the final model, we train the model using values for p∈{2,3,…,P}. RF and XGB models are implemented in R using the ‘caret’, ‘randomForest’, and ‘xgboost’ packages. For the XGB model, we focus on tuning the learning rate (η), $L_{2}$ regularization penalty (λ), and maximum tree depth, while for RF model, the number of trees (ntree) and the maximum number of terminal nodes (maxnodes) are tuned. All the optimizations are based on achieving the maximum percentage agreement values. After identifying the optimal lag parameter ($p^{*}$) and hyperparameters, the final model is trained using the data up to $y_{T,i}$ for each region i. Once the final model is fitted, h-step ahead forecasts are generated through a recursive forecasting method.

The steps are as follows:

1. Initial forecast at T+1:
  - Construct the feature vector for T+1 using the last $p^{*}$ values:
$$X_{T+1,i}=[y_{T-p^{*}+1,i}\;y_{T-p^{*}+2,i}\;\cdots \;y_{T-1,i}\;y_{T,i}\;D_{T+1,i}].$$
  - Predict the value at T+1 using the fitted model:
$${\hat{y}}_{T+1,i}=X_{T+1,i}\;\hat{\beta }.$$
2. Update lagged features:
  - Append the forecasted value ${\hat{y}}_{T+1,i}$ to the time series. Discard the oldest value ($y_{T-p^{*}+1,i}$) tom maintain $p^{*}$ lagged values for the next step. The updated feature vector is:
$$X_{T+2,i}=[y_{T-p^{*}+2,i}\;y_{T-p^{*}+3,i}\;\cdots \;y_{T,i}\;{\hat{y}}_{T+1,i}\;D_{T+2,i}].$$
  - Predict the value at T+1 using the fitted model:
$${\hat{y}}_{T+2,i}=X_{T+2,i}\;\hat{\beta }.$$
3. Iterates for h forecast horizon:
  Repeat the process in step-2 until forecast ${\hat{y}}_{T+1,i},{\hat{y}}_{T+2,i},\ldots ,{\hat{y}}_{T+h,i}$ are obtained for each region i.

For forecasting using the initial estimates ${\hat{R}}_{t,i}$, we employ Reg, RF, and XGB models, with the forecasted values represented as ${\hat{R}}_{t,\text{Reg},i}^{F},{\hat{R}}_{t,\text{RF},i}^{F}$, and ${\hat{R}}_{t,\text{XGB},i}^{F}$, respectively. Similarly, when using the spatially smoothed estimates ${\hat{R}}_{t,i}^{S}$, we apply the same methods, with the forecasted values denoted as ${\hat{R}}_{t,\text{Reg},i}^{\text{SF}},{\hat{R}}_{t,\text{RF},i}^{\text{SF}}$, and ${\hat{R}}_{t,\text{XGB},i}^{\text{SF}}$, respectively.

For each forecasting method, we follow the steps outlined above (Steps 1–3). The forecasting models are trained using lagged values of $R_{t}$ estimates and relevant temporal features. Once fitted, the models generate forecasts recursively, where predicted values are iteratively used as inputs for subsequent forecasts. This process is repeated until the specified forecasting horizon is achieved.

Finally, we implement an ensemble-based forecasting approach that integrates all individual forecasts. The weight assigned to each forecasting method is determined using the percentage agreement (PA) metric, calculated using equation (1) in section 2.5, which evaluates the accuracy of each method by comparing its forecasts with ${\hat{R}}_{t,i}^{S}$. The relative proportion of PA for each method is then used to determine its contribution to the ensemble forecast. The resulting ensemble-based forecast is denoted as ${\hat{R}}_{t,\text{Ensemble},i}^{F}$.

### 2.4 Forecasting COVID-19 daily cases

To forecast the COVID-19 daily cases, we utilize a probabilistic framework that incorporates the serial interval distribution, historical case data, and forecasted effective reproductive number, ${\hat{R}}_{t,i}^{F}$. We use ${\hat{R}}_{t,i}^{F}$ as a general term to describe the case forecasting steps; however, case forecasts are generated for each individual $R_{t}$ forecasts, including ${\hat{R}}_{t,Reg,i}^{F},{\hat{R}}_{t,\text{RF},i}^{F},{\hat{R}}_{t,\text{XGB},i}^{F},{\hat{R}}_{t,Reg,i}^{\text{SF}},{\hat{R}}_{t,\text{RF},i}^{\text{SF}}$, and ${\hat{R}}_{t,\text{XGB},i}^{\text{SF}}$, as well as for the ensemble forecast, ${\hat{R}}_{t,\text{Ensemble},i}^{F}$. The corresponding case forecast from these different $R_{t}$ estimates are presented as ${\hat{I}}_{t,Reg,i},{\hat{I}}_{t,\text{RF},i},{\hat{I}}_{t,\text{XGB},i},{\hat{I}}_{t,Reg,i}^{S},{\hat{I}}_{t,\text{RF},i}^{S},{\hat{I}}_{t,\text{XGB},i}^{S}$ and ${\hat{I}}_{t,\text{Ensemble},i}$.

The serial interval, representing the time between symptom onset in a primary case and secondary case, is assumed to follow a gamma distribution. This distribution is used to compute the total infectiousness ($\Lambda_{t,i}$) for each day t at region i, which quantifies the potential of previously observed cases to generate new infections. Total infectiousness is calculated as:

$$\Lambda_{t,i}=\sum_{k=1}^{s}I_{t-k,i}\cdot p_{k}$$

where $I_{t-k,i}$ is the number of observed cases on day t−k at location $i,p_{k}$ is the probability that a primary case generates a secondary case between k−1 and k days. The term $\Lambda_{t,i}$ captures the cumulative infections potential of cases reported in the preceding s days, weighted by the serial interval distribution.

Using the forecasted effective reproductive number (${\hat{R}}_{t,i}^{F}$) for day t, the expected number of cases ($\text{E}[I_{t,i}]$) is calculated as:

$$\text{E}[I_{t,i}]=\Lambda_{t,i}\times {\hat{R}}_{t,i}^{F}$$

Daily case counts are then modeled as a Poisson random variable:

$$I_{t,i}\sim \text{Poisson}(\Lambda_{t,i}\times {\hat{R}}_{t,i}^{F})$$

The COVID-19 case forecasts are generated using a recursive approach similar to that used for obtaining ${\hat{R}}_{t,i}^{F}$. The main steps are as follows:

1. Initial forecast (t=T+1):
  - Compute $\Lambda_{T+1,i}$ using historical case data up to day T fore region i.
  - Generate N samples from Poisson distribution:
$$I_{T+1,i}^{\left(n\right)}\sim \text{Poisson}(\Lambda_{T+1,i}\times {\hat{R}}_{T+1,i}^{F}),n=1,2,\ldots ,N.$$
  - Obtain ${\hat{I}}_{T+1,i}$ by computing the average of the samples.
$${\hat{I}}_{T+1,i}=\frac{1}{N}\sum_{n=1}^{N}I_{T+1,i}^{\left(n\right)}$$
  - Append ${\hat{I}}_{T+1,i}$ to the historical case data.
2. Iterative case forecast (t=T+2,T+3,…,T+h):
  For each subsequent day, repeat the following steps:
  - Calculate $\Lambda_{t,i}$ using updated historical and forecasted case data:
$$\Lambda_{t,i}=\sum_{k=1}^{s}I_{t-k,i}\cdot p_{k}$$
  - Draw N samples from the Poisson distribution:
$$I_{t,i}^{\left(n\right)}\sim \text{Poisson}(\Lambda_{t,i}\times {\hat{R}}_{t,i}^{F}),k=1,2,\ldots ,K.$$
  - Compute the mean of N samples to obtain case forecast, ${\hat{I}}_{t,i}$
$${\hat{I}}_{t,i}=\frac{1}{N}\sum_{n=1}^{N}I_{t,i}^{\left(n\right)}$$
  - Append ${\hat{I}}_{t,i}$ to the historical case data.
3. Continue this process to generate forecasts for h forecast horizon (${\hat{I}}_{T+1,i},{\hat{I}}_{T+2,i},\ldots ,{\hat{I}}_{T+h,i}$).

We generate 1000 samples from the Poisson model and compute the average as the case forecast for each step. The accuracy of the case forecasts is assessed by computing the median percentage agreement along with the interquartile range (IQR) across all 46 counties in SC.

### 2.5 Performance evaluation of $R_{t}$ and case forecasts

To assess the performance of forecasting models for both the effective reproductive number ($R_{t}$) and COVID-19 daily cases, we use percentage agreement (PA).

Let $O_{t,i}$ denotes the observed values at region i and time t (e.g. daily cases, we do not know observed values for $R_{t,i}$, use estimates ${\hat{R}}_{t,i}^{S}$), $P_{t,i}$ represents the forecasted value at region i and time t (e.g. ${\hat{R}}_{t,\text{Reg},i}^{F},{\hat{R}}_{t,\text{RF},i}^{F},{\hat{R}}_{t,\text{XGB},i}^{F},{\hat{R}}_{t,\text{Reg},i}^{\text{SF}},{\hat{R}}_{t,\text{RF},i}^{\text{SF}},{\hat{R}}_{t,\text{XGB},i}^{\text{SF}},{\hat{R}}_{t,\text{Ensemble},i}^{F}$ and ${\hat{I}}_{t,\text{Reg},i},{\hat{I}}_{t,\text{RF},i},{\hat{I}}_{t,\text{XGB},i},{\hat{I}}_{t,Reg,i}^{S},{\hat{I}}_{t,\text{RF},i}^{S},{\hat{I}}_{t,\text{XGB},i}^{S},{\hat{I}}_{t,\text{Ensemble},i}$). Then the evaluation metrics are defined as follows:

Percentage Agreement (PA):

$$\text{PA}=\frac{1}{T}\sum_{t=1}^{T}\frac{\text{min}(O_{t,i},\;P_{t,i})}{\text{max}(O_{t,i},\;P_{t,i})}\times 100$$

For the $R_{t}$ forecast, we compute pointwise PA, meaning that the agreement is assessed for each time point t, followed by averaging the PA values over the entire forecasting period. For the case forecast, we compute weekly aggregated PA, where observed and forecasted cases are summed over consecutive 7-day periods, and PA is calculated based on these aggregated weekly totals before averaging across the forecasting period.

All model training and evaluations are conducted using R software on a computing system with processor: 13th Gen Intel(R) Core (TM) i7-13700 2.10 GHz; RAM: 32 GB; System: 64-bit operating system, x64-based processor. We use EpiNow2 R package version 1.4.0, with seed = 100 for all analyses to ensure reproducibility. For forecasting during Scenario-1 and Scenario-2, we optimize lagged features and tuned hyperparameters for Reg, RF, and XGB models, applied to both initial estimates (${\hat{R}}_{t,\text{Now},i}$) and spatially smoothed estimates (${\hat{R}}_{t,\text{Now},i}^{S}$). We perform 5-fold cross-validation for optimal lag selection and hyperparameter tuning. A detailed summary of the selected lagged features and model-specific hyperparameters is provided in the supplementary materials (Table S1).

## 3 Results

### 3.1 Evaluation of $R_{t}$ forecasting performance

The median percentage agreement (PA) across counties for ensemble-based forecasts was consistently higher than that of EpiNow2 (Table 1). For Scenario-1, the ensemble method achieved a median PA across counties of 94.4% (IQR: 93.8% – 95.3%) for 7-day ahead forecasts, while EpiNow2 reached 87.0% (IQR: 84.4% – 89.4%). Similarly, in Scenario-2, the ensemble approach maintained a median PA of 93.0% (IQR: 91.3% % – 94.1%) for 7-day ahead forecasts compared to a median PA of 86.8% (IQR: 82.5% – 89.2%) for EpiNow2.

Table 1 provides a detailed comparison of forecasting accuracy for 7-day, 14-day, and 21-day ahead of forecasts for 84 days period during both Scenario-1 and Scenario-2. The ensemble-based approach consistently outperformed EpiNow2 across all forecast horizons. The relative difference in the 7-day ahead percentage agreement was 8.5% higher for the ensemble approach in the first forecast period (94.4% vs 87.0%) and 7.1% higher in the second forecasting period (93.0% vs. 86.8%). While the percentage agreement decreased as the forecast horizon increased, the ensemble method retained higher predictive accuracy compared to EpiNow2. The relative difference in 21-day ahead percentage agreement was 5.7% higher for the ensemble approach in the first forecast period (93.0% vs 87.6%) and 6.2% higher in the second forecasting period (91.9% vs 86.5%).

In counties with higher COVID-19 case counts (Figure-1, left panel): Charleston, Greenville, Horry, Lancaster, Lexington, York), both methods captured trends well, but EpiNow2 (red lines) exhibited more fluctuations, particularly in Horry and Lexington, where large spikes deviated significantly from the general trend. The ensemble model (blue lines) provided smoother forecasts, aligning more closely with the spatially smoothed $R_{t}$ estimates (black dashed lines), indicating a more stable performance in capturing transmission trends. In counties with lower COVID-19 case counts (Figure-1, right panel): Allendale, Bamberg, Barnwell, Edgefield, McCormick, Saluda), the ensemble approach still performed consistently better, but EpiNow2 showed even more fluctuations, especially in Edgefield. The ensemble model produced smoother and more consistent forecasts, maintaining stability despite lower case counts.

Across all counties, the ensemble approach consistently provided higher agreement with observed trends, while EpiNow2 showed more variability, particularly in some counties with significant fluctuations (e.g., Horry and Edgefield). The supplementary materials provide further insights into forecast performance across all counties. Figure S1 presents 7-day ahead $R_{t}$ forecasts for all 46 counties, while Figures S2 and S3 display the 14-day and 21-day ahead forecasts, respectively, for Scenario-1. Similarly, Figures S4–S6 provide the 7-day, 14-day, and 21-day forecasts for Scenario-2, reaffirming that the ensemble model consistently outperformed EpiNow2, particularly in regions with highly variable transmission dynamics.

Figure S7 presents the 7-day ahead spatially smoothed $R_{t}$ forecasts from EpiNow2 alongside ensemble forecasts and spatially smoothed $R_{t}$ estimates for all 46 counties in SC during Scenario-1. Table S2 summarizes the forecast accuracy for $R_{t}$ in both Scenario-1 and Scenario-2 based on comparisons with the initial estimates of $R_{t}$, while Figure S8 provides an illustration of the forecast results. The results show that ensemble-based forecasts consistently outperformed EpiNow2 forecasts across both scenarios and forecast horizons, with ensemble models achieving median PA 93.5% for 7-day ahead forecasts in Scenario-1 and 89.8% in Scenario-2. These correspond to approximately 9.1% and 1.4% higher relative performance compared to EpiNow2 forecasts, respectively.

### 3.2 Evaluation of COVID-19 case forecasting performance

The results indicated that the ensemble approach consistently outperformed EpiNow2, particularly for short-term forecasts (7-day and 14-day horizons). In Scenario-1, the ensemble model achieved a median PA of 85.7% (IQR: 83.6% – 88.6%) for 7-day forecasts, slightly outperforming EpiNow2, which had a median PA of 83.8% (IQR: 81.6% – 86.9%). For longer horizons (21-day forecasts), the ensemble approach maintained a median PA of 81.0% (IQR: 77.5% – 83.4%), while EpiNow2 showed a lower median PA of 67.6% (IQR: 61.3% – 75.1%), demonstrating that the ensemble method provided greater stability in extended forecasting periods.

A similar trend was observed in Scenario 2, where ensemble-based forecasts continued to outperform EpiNow2 across all forecast horizons. The relative improvement in percentage agreement was more evident for longer-term predictions, with the ensemble model achieving a median PA of 74.8% (IQR: 67.5% – 78.9%) for 21-day forecasts, compared to 66.2% (IQR: 57.4% – 73.2%) for EpiNow2. Table 2 presents a detailed comparison of forecast accuracy metrics for 7-day, 14-day, and 21-day ahead predictions across both scenarios. The ensemble-based method consistently demonstrated higher predictive accuracy, particularly for longer forecast horizons, where EpiNow2’s performance declined more significantly.

The results indicate that the ensemble approach produced more stable and accurate case forecasts than EpiNow2, with differences varying by county and case count levels (Figure 3). In higher case count counties (Charleston, Greenville, Horry, Lancaster, Lexington, York), the ensemble model captured trends more smoothly, whereas EpiNow2 exhibited abrupt spikes and larger deviations from observed case counts, particularly in Horry and Lexington. In lower case count counties (Allendale, Bamberg, Barnwell, Edgefield, McCormick, Saluda), the performance gap between the two methods was more evident in certain areas, such as Edgefield, where EpiNow2 produced inflated peaks not reflected in observed data.

Overall, the results confirm that the ensemble forecasting approach provided more accurate and stable case forecasts across all time horizons and geographical regions. The ensemble forecasts consistently maintained higher median percentage agreements, particularly for longer-term forecasts (21-day ahead), where EpiNow2’s accuracy declined significantly. The supplementary materials provide a comprehensive summary of forecast performance across all counties. Figures S9–S11 (Scenario-1) and Figures S12–S14 (Scenario-2) present case forecasts for all counties at 7-day, 14-day, and 21-day ahead horizons, respectively. These figures further illustrate the ensemble model’s advantage in reducing forecast variability and improving agreement with observed case counts

### 3.2 $R_{t}$ and COVID-19 case forecasting performance of individual models

Table S3 indicates that RF models consistently achieved higher median PA values compared to Reg and XGB models, especially when trained on spatially smoothed estimates. For instance, during Scenario-1, RF models with spatial smoothing attained a median PA of 96.5% (IQR: 96.0%–97.0%) for 7-day ahead forecasts, whereas Reg and XGB models with spatial smoothing had median PAs of 94.1% (IQR: 92.9%–94.9%) and 93.2% (IQR: 92.1%–94.2%), respectively. EpiNow2 with spatial smoothing obtained a median PA of 87.6% (IQR: 86.6%–88.9%).

As shown in Table S4, the XGB model with spatial smoothing outperformed all the other individual models in forecasting daily COVID-19 cases. In Scenario-1, XGB with spatial smoothing achieved a median PA of 86.5% (IQR: 84.4%–88.6%) for 7-day ahead forecast, surpassing Reg and RF models with spatial smoothing, which had median PAs of 85.9% (IQR: 84.1%–88.4%) and 86.0% (IQR: 82.8%–88.1%), respectively.

The forecasting performance of Reg, RF, and XGB models is evaluated through Figures S15–S20 for $R_{t}$ forecasts and Figures S21–S26 for COVID-19 case forecasts, across different forecast horizons. For $R_{t}$ forecasting, all Reg, RF, and XGB models consistently provided predictions, closely following the smoothed $R_{t}$ estimates across different counties. For COVID-19 case forecasting in Scenario-1, XGB and RF with spatial smoothing outperformed the other models, particularly in counties with complex transmission dynamics. In Scenario-2 case forecasting, XGB and RF without spatial smoothing achieved higher prediction accuracy compared to the other models.

## 4 Discussion

Accurate forecasting of $R_{t}$ and COVID-19 case counts is critical for epidemic control and public health decision-making. Previous studies have demonstrated the importance of real-time $R_{t}$ estimation in outbreak control, emphasizing its role in quantifying disease transmissibility and guiding policy responses (Gostic et al., 2020; Parag, 2021). Traditional statistical models, such as those used in Bayesian inference-based approaches like EpiNow2, have been widely employed to estimate $R_{t}$ in real time (Abbott et al., 2021; Gostic et al., 2020). This study integrates machine learning techniques and a probabilistic framework to address the challenges of forecasting disease transmission and cases. The integration of machine learning methods into epidemic forecasting has shown promise in improving predictive accuracy and adaptability (Athanasios et al., 2021; Caruso et al., 2022; Song et al., 2022; Uddin et al., 2019). Our findings indicate that the ensemble-based approach consistently outperformed the widely used EpiNow2 model across different forecasting horizons. Several studies have also shown that integrating multiple models enhances predictive robustness, as it leverages different model strengths to mitigate biases inherent in individual approaches (Lal et al., 2021; Oidtman et al., 2021, 2021; Reich et al., 2019).

The practical implications of our forecasting framework are significant. Accurate $R_{t}$ predictions enable early detection of outbreaks, allowing for timely interventions such as targeted lockdowns or vaccination campaigns. Similarly, reliable case forecasts inform resource allocation, ensuring that medical supplies, hospital beds, and testing infrastructure are optimally distributed. The ability to evaluate policy effectiveness through changes in $R_{t}$ and forecasted trends supports evidence-based decision-making. Moreover, transparent and reliable forecasts enhance public trust and compliance with health guidelines, countering misinformation and fostering collective action. Collaborations between epidemiologists, policymakers, and data scientists can facilitate the deployment of automated forecasting systems for real-time outbreak monitoring (Lison et al., 2022).

While the proposed forecasting framework demonstrated improved accuracy and robustness compared to EpiNow2, several limitations must be acknowledged. Data quality and availability remain key challenges, such as reporting delays, underreporting, and inconsistencies in case data can introduce biases in estimating infectiousness and subsequent forecasts. A major limitation is the lack of more granular forecasts, such as at the ZIP code level, which could enhance localized public health interventions. Previous studies have shown that geographically refined predictions can better inform targeted responses, including mobile health clinic (MHC) deployments (Gezer et al., 2024). More detailed spatial forecasts would allow public health officials to allocate resources more effectively, particularly in underserved communities where healthcare access is limited. Additionally, real-time forecasting remains challenging due to potential delays in obtaining case data from state health departments. In practice, alternative data sources, such as electronic health records (EHR) from health systems, may be necessary to supplement official reports. Previous research has demonstrated promise in leveraging alternative data sources for predictive modeling, but adapting models for real-time implementation in such settings requires further validation (Gezer et al., 2024). Furthermore, forecast accuracy declines as the prediction horizon increases, a common issue in time series forecasting. While the ensemble approach mitigates some of this decline, 21-day forecasts exhibit greater variability and lower percentage agreement compared to shorter forecasts.

Future research could focus on integrating Bayesian deep learning techniques with real-time data streams, such as mobility, genomic surveillance, wastewater data, and electronic health records, to improve forecast accuracy and uncertainty quantification. Developing adaptive and explainable models that dynamically update with new data will enhance their utility for real-time public health decision-making.

## Conclusion

In this study, we developed a forecasting framework to predict the effective reproductive number ($R_{t}$) and COVID-19 daily case counts using a combination of machine learning models and probabilistic approaches. By leveraging Bayesian estimates, spatial smoothing, and recursive forecasting, our approach enhances the accuracy of epidemic predictions. The ensemble-based methodology further refines forecast by optimally combining individual model outputs using percentage agreement as a weighting criterion. Our results demonstrate that incorporating spatially adjusted estimates improves forecast performance across different pandemic waves and forecasting horizons. While the framework shows strong predictive capability, some limitations remain. The accuracy of forecasts depends on the quality and timeliness of reported case data. Future work should explore adaptive modeling techniques that integrate real-time mobility data and genomic surveillance to refine predictions further. Additionally, expanding the framework to accommodate multiple infectious diseases could enhance its applicability for broader epidemiological forecasting. Overall, this study contributes to advancing epidemic forecasting by integrating machine learning and probabilistic models in a flexible and scalable framework. These findings can support data-driven policy interventions, improve epidemic preparedness, and facilitate more effective public health responses in future outbreaks.

## Supplementary Material

1

## Figures and Tables

**Figure 2.**
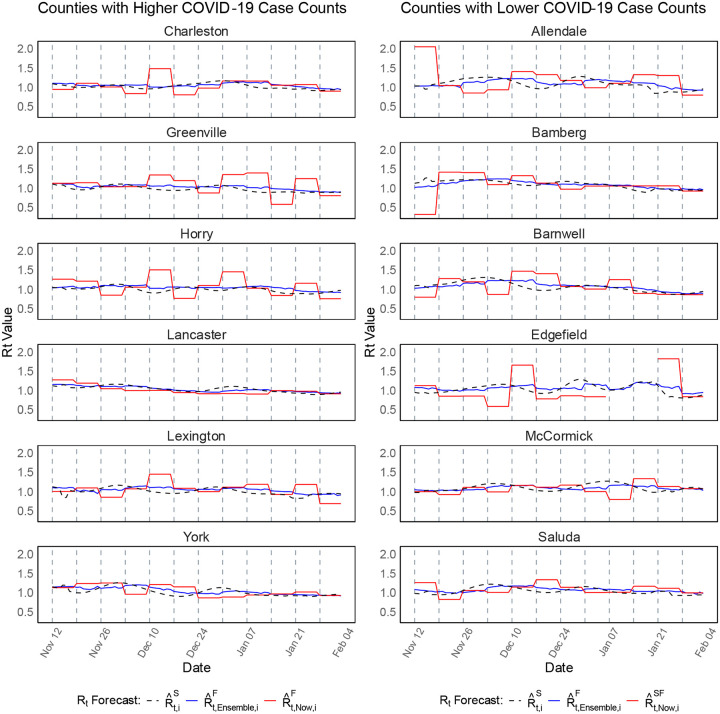
Forecast of $R_{t}$ at the county level in SC during Scenario-1 (November 11, 2020 – February 02, 2021). The left panel presents $R_{t}$ forecasts for six counties with higher COVID-19 case counts, while the right panel displays forecasts for six counties with lower case counts. The plots compare the ensemble-based forecasts (blue lines) with those generated using the **EpiNow2** R package (red lines), alongside the spatially (covariate-adjusted) smoothed estimates, ${\hat{\text{R}}}_{\text{t},\text{i}}^{\text{S}}$ (black dashed lines), where i presents the county, t denotes the time point (day), and “Now” refers to the **EpiNow2** method. Forecasts were generated using a rolling window approach for 7-day ahead predictions over 84 days period.

**Figure 03.**
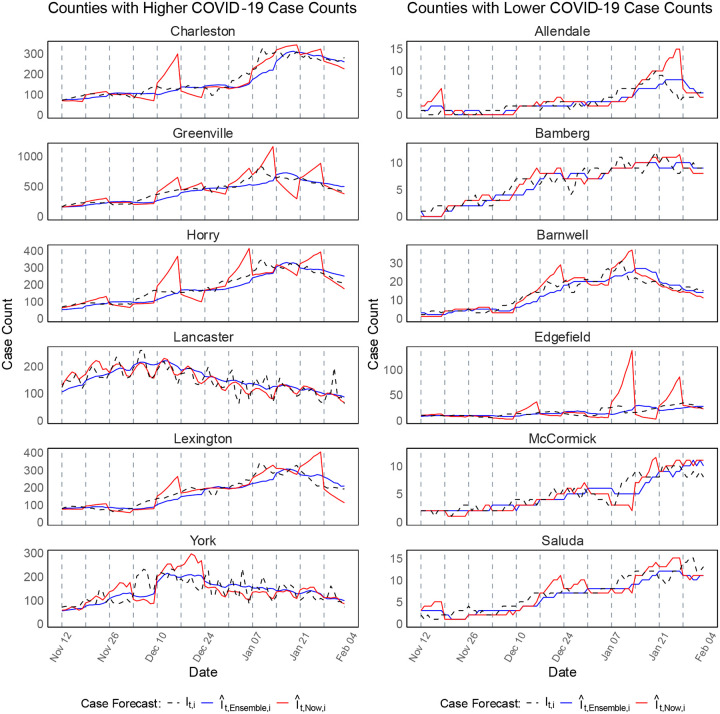
Forecast of COVID-19 case counts at the county level in SC during Scenario-1 (November 11, 2020 – February 02, 2021). The left panel presents forecasts for six counties with higher COVID-19 case counts, while the right panel displays forecasts for six counties with lower case counts. The plots compare EpiNow2 forecasts (red lines) and ensemble-based forecasts (blue lines) against the observed daily case counts (black dashed lines). The forecasts were generated for 7-day ahead predictions over 84 days period using a rolling window approach.

**Table 01.** Forecast accuracy metrics for the effective reproductive number (${\text{R}}_{\text{t}}$) in SC counties during two forecast periods: November 11, 2020 – February 02, 2021 (Scenario-1) and December 11, 2022 – March 04, 2023 (Scenario-2). Forecast models were trained with data from June 01, 2020, to November 10, 2020, for Scenario-1, and from May 01, 2022, to December 10, 2022, for Scenario-2. The forecasting approach employed a rolling window method for 7-day, 14-day, and 21-day ahead predictions over 84 days period. The table presents the median percentage agreement (PA) across counties along with the interquartile range (IQR) of county PAs.

Forecast Method	Effective Reproductive Number Forecast: Percentage Agreement (PA), Median (IQR)					
	Forecast Period: November 11, 2020 – February 02, 2021 (Scenario-1)			Forecast Period: December 11, 2022 – March 04, 2023 (Scenario-2)		
	7-day ahead	14-day ahead	21-day ahead	7-day ahead	14-day ahead	21-day ahead
EpiNow2	87.0% (84.4% – 89.4%)	86.2% (81.5% – 89.5%)	87.6% (85.1% – 90.6%)	86.8% (82.5% – 89.2%)	86.7% (81.4% – 89.9%)	86.5% (79.5% – 89.6%)
Ensemble	94.4% (93.8% – 95.3%)	93.0% (92.0% – 94.3%)	92.6% (91.6% – 93.7%)	93.0% (91.3% – 94.1%)	92.7% (90.9% – 93.9%)	91.9% (89.8% – 93.1%)

**Table 02.** Forecast accuracy metrics for COVID-19 daily case counts at the county level in SC during Scenario 1 (November 11, 2020 – February 2, 2021) and Scenario 2 (December 11, 2022 – March 4, 2023). Forecast accuracy was evaluated using percentage agreement (PA) with median and interquartile range (IQR) across all 46 counties. Case forecasts were generated using the EpiNow2 R package and a probabilistic Poisson model based on the ensemble-based $R_{t}$ forecasts (${\hat{\text{R}}}_{\text{t},\text{Ensemble},\text{i}}^{\text{F}}$). Forecasts were conducted for 7-day, 14-day, and 21-day ahead predictions over 84 days period using a rolling window approach.

Forecast Method	COVID-19 Daily Cases Forecast: Percentage Agreement (PA), Median (IQR)					
	Forecast Period: November 11, 2020 – February 02, 2021 (Scenario-1)			Forecast Period: December 11, 2022 – March 04, 2023 (Scenario-2)		
	7-day ahead	14-day ahead	21-day ahead	7-day ahead	14-day ahead	21-day ahead
EpiNow2	83.8% (81.6% – 86.9%)	75.4% (72.4% – 78.7%)	67.6% (61.3% – 75.1%)	75.1% (67.5% – 79.1%)	73.5% (65.3% – 76.7%)	66.2% (57.4% – 73.2%)
Ensemble	85.7% (83.6% – 88.6%)	84.2% (80.8% – 86.9%)	81.0% (77.5% – 83.4%)	78.4% (75.2% – 81.7%)	77.0% (72.8% – 80.3%)	74.8% (67.5% – 78.9%)

## Data Availability

All code and public data used for analyses and figure generations are available at https://github.com/mdsakhh/Machine-Learning-and-Probabilistic-Approach-for-Rt-and-COVID-19-Case-Forecasting and from the corresponding author upon request.
