# Supplementary material for: Machine Learning and Probabilistic Approaches for Forecasting COVID-19 Transmission and Cases
Source: medRxiv. 2025 Jun 24:2025.06.24.25330210. Preprint. [Version 1] doi: 10.1101/2025.06.24.25330210 (PMC12262794; doi:10.1101/2025.06.24.25330210)
Supplement: 1 [file NIHPP2025.06.24.25330210V1-supplement-1.pdf]

medRxiv preprint doi: https://doi.org/10.1101/2025.06.24.25330210; this version posted June 24, 2025. The copyright holder for this preprint (which was not certified by peer review) is the author/funder, who has granted medRxiv a license to display the preprint in perpetuity.
It is made available under a CC-BY 4.0 International license .

Supplementary Materials:

Table S1. Summary of optimal lag (lagged feature) selection and hyperparameter tuning for Regression, Random Forest, and XGBoost models in Scenario-1 (November 11, 2020 – February 02, 2021) and Scenario-2 (December 11, 2022 – March 04, 2023), based on 5-fold cross-validation.

Forecast Period	Parameters	Regression	Regression (Smooth)	RF	RF (Smooth)	XGBoost	XGBoost (Smooth)
**Scenario-1**	optimal lag	12	4	10	3	18	7
	L_2 penalty (λ)	---	---	---	---	3	5
	learning rate (η)	---	---	---	---	0.05	0.05
	max depth	---	---	---	---	8	10
	node size	---	---	10	10	---	---
	ntree	---	---	500	400	---	---
	maxnodes	---	---	150	150	---	---
**Scenario-2**	optimal lag	19	19	3	3	14	24
	L_2 penalty (λ)	---	---	---	---	2	1
	learning rate (η)	---	---	---	---	0.05	0.05
	max depth	---	---	---	---	8	8
	node size	---	---	10	10	---	---
	ntree	---	---	200	500	---	---
	maxnodes	---	---	150	150	---	---

medRxiv preprint doi: https://doi.org/10.1101/2025.06.24.25330210; this version posted June 24, 2025. The copyright holder for this preprint
(which was not certified by peer review) is the author/funder, who has granted medRxiv a license to display the preprint in perpetuity.
It is made available under a CC-BY 4.0 International license .

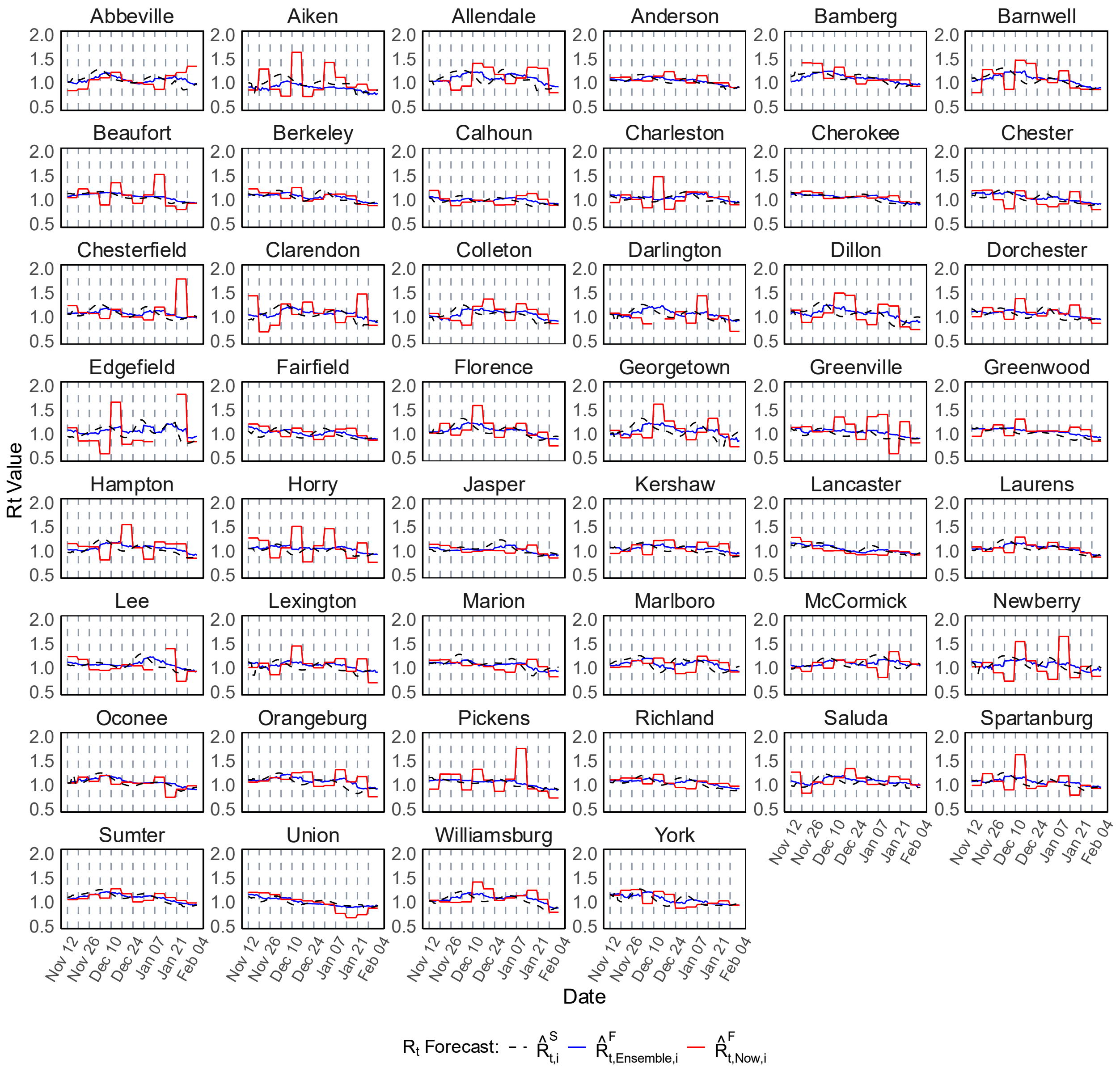

Figure S1. Forecast of R_t at the county level in SC during Scenario-1 (November 11, 2020 – February 02, 2021). This figure presents the forecast of R_t for all 46 counties in SC. The plots compare the ensemble-based forecast (blue lines) and forecasts generated using the EpiNow2 R package (red lines) alongside the spatially (covariate-adjusted) smoothed estimates ($\widehat{R}^S_{t,Now,i}$, black dashed lines), where i represents the county, t denotes the time point (day), and "Now" refers to the EpiNow2 method. The forecasts were generated for 7-day ahead predictions over 84 days period using a rolling window approach.

medRxiv preprint doi: https://doi.org/10.1101/2025.06.24.25330210; this version posted June 24, 2025. The copyright holder for this preprint (which was not certified by peer review) is the author/funder, who has granted medRxiv a license to display the preprint in perpetuity.
It is made available under a CC-BY 4.0 International license .

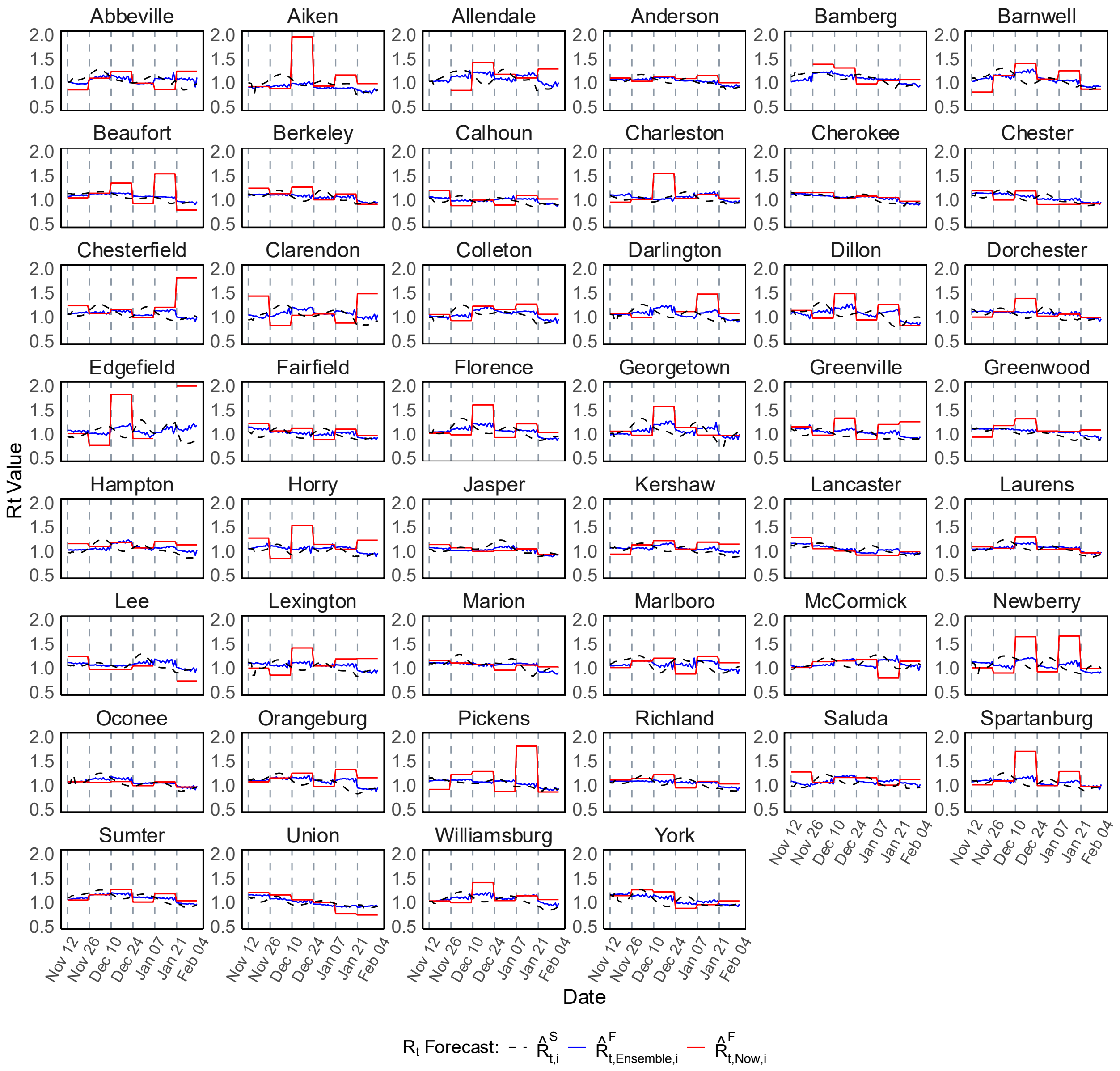

Figure S2. Forecast of R_t at the county level in SC during Scenario-1 (November 11, 2020 – February 02, 2021). This figure presents the forecast of R_t for all 46 counties in SC. The plots compare the ensemble-based forecast (blue lines) and forecasts generated using the EpiNow2 R package (red lines) alongside the spatially (covariate-adjusted) smoothed estimates ($\widehat{R}^{S}_{t,Now,i}$, black dashed lines), where i represents the county, t denotes the time point (day), and "Now" refers to the EpiNow2 method. The forecasts were generated for 14-day ahead predictions over 84 days period using a rolling window approach.

medRxiv preprint doi: https://doi.org/10.1101/2025.06.24.25330210; this version posted June 24, 2025. The copyright holder for this preprint (which was not certified by peer review) is the author/funder, who has granted medRxiv a license to display the preprint in perpetuity.
It is made available under a CC-BY 4.0 International license .

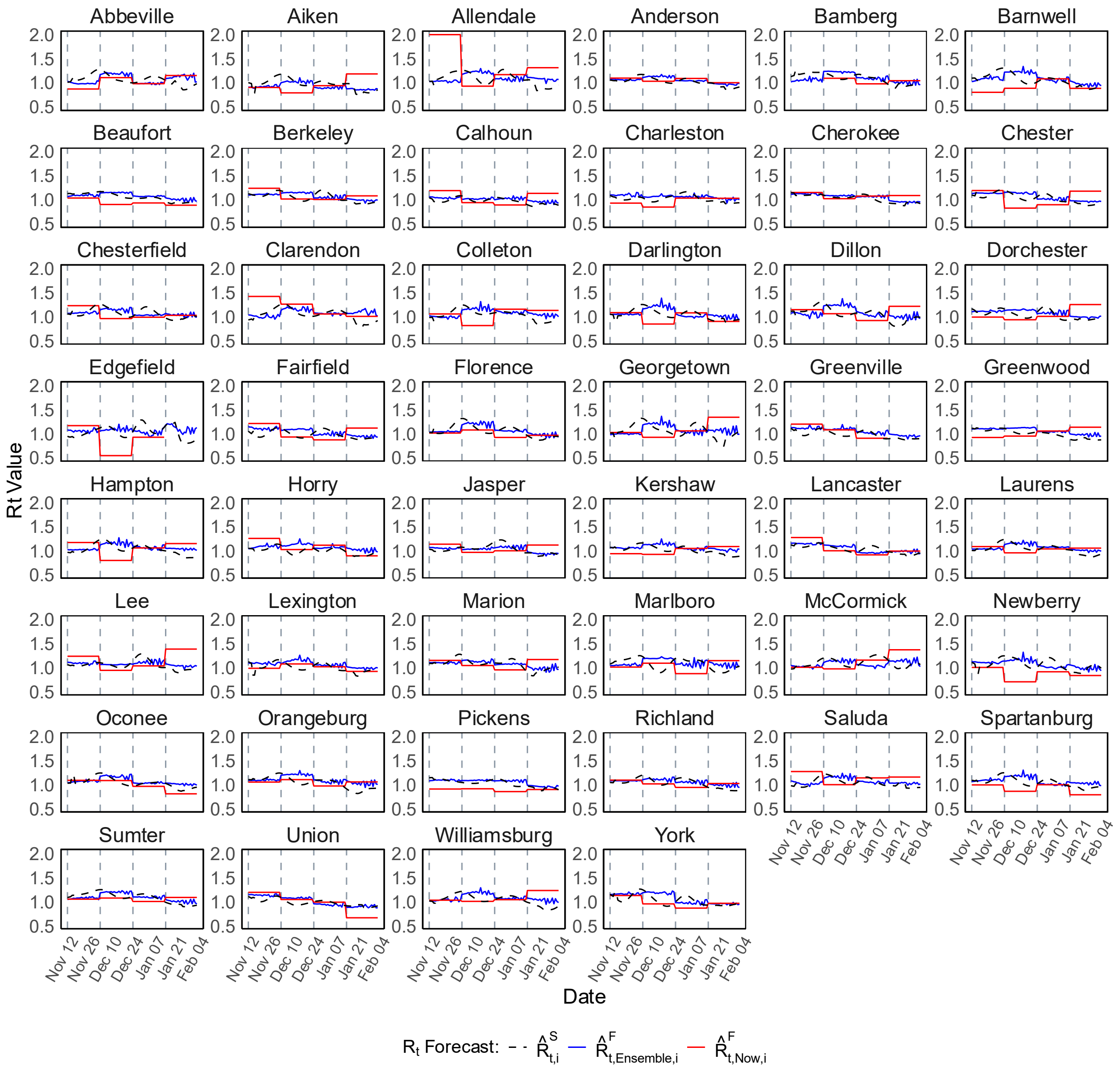

Figure S3. Forecast of R_t at the county level in SC during Scenario-1 (November 11, 2020 – February 02, 2021). This figure presents the forecast of R_t for all 46 counties in SC. The plots compare the ensemble-based forecast (blue lines) and forecasts generated using the EpiNow2 R package (red lines) alongside the spatially (covariate-adjusted) smoothed estimates ($\widehat{R}^{S}_{t,Now,i}$, black dashed lines), where i represents the county, t denotes the time point (day), and "Now" refers to the EpiNow2 method. The forecasts were generated for 21-day ahead predictions over 84 days period using a rolling window approach.

medRxiv preprint doi: https://doi.org/10.1101/2025.06.24.25330210; this version posted June 24, 2025. The copyright holder for this preprint (which was not certified by peer review) is the author/funder, who has granted medRxiv a license to display the preprint in perpetuity.
It is made available under a CC-BY 4.0 International license .

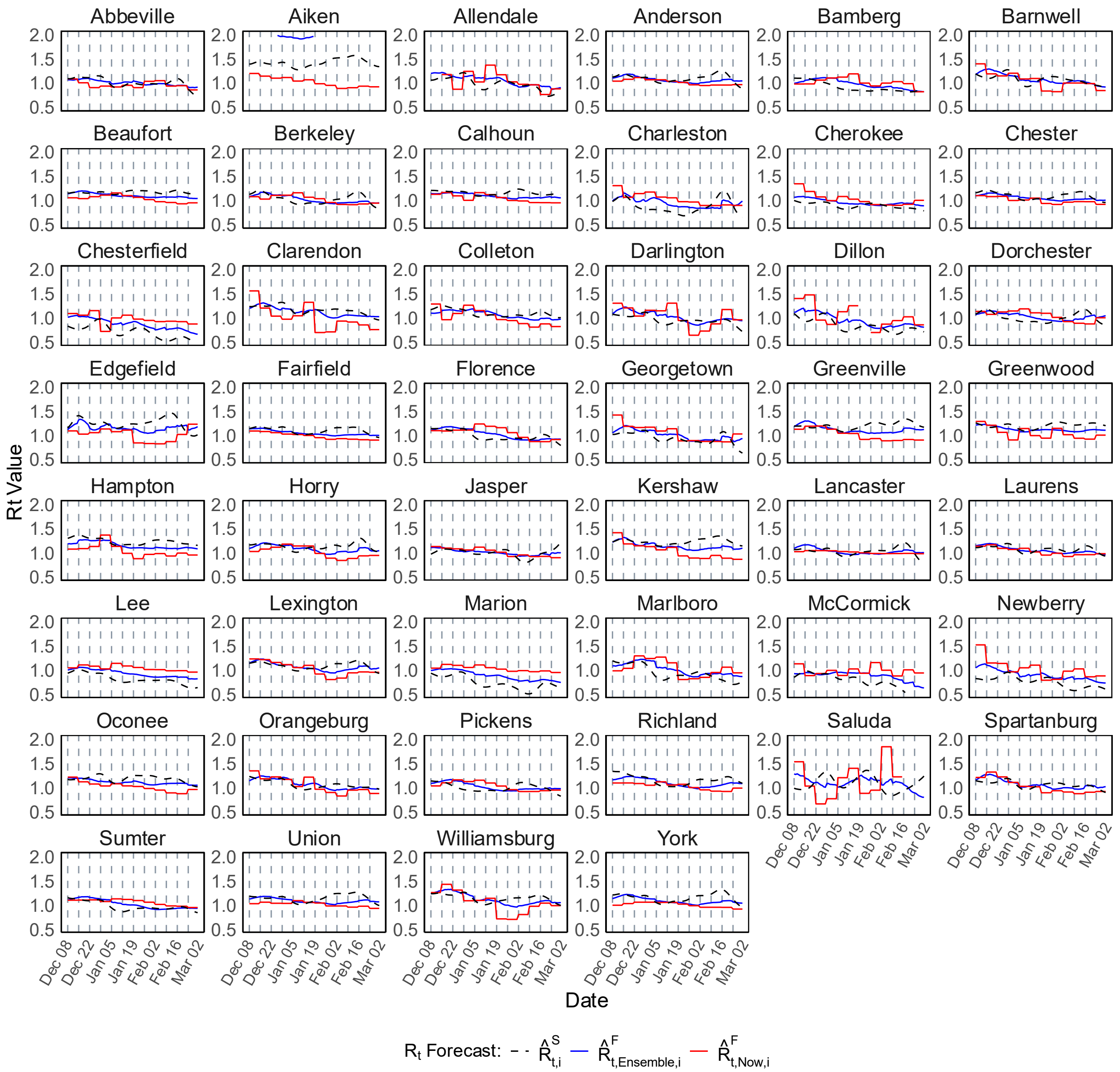

Figure S4. Forecast of R_t at the county level in SC during Scenario-2 (December 11, 2022 – March 04, 2023). This figure presents the forecast of R_t for all 46 counties in SC. The plots compare the ensemble-based forecast (blue lines) and forecasts generated using the EpiNow2 R package (red lines) alongside the spatially (covariate-adjusted) smoothed estimates ($\widehat{R}^S_{t,Now,i}$, black dashed lines), where i represents the county, t denotes the time point (day), and "Now" refers to the EpiNow2 method. The forecasts were generated for 7-day ahead predictions over 84 days period using a rolling window approach.

medRxiv preprint doi: https://doi.org/10.1101/2025.06.24.25330210; this version posted June 24, 2025. The copyright holder for this preprint (which was not certified by peer review) is the author/funder, who has granted medRxiv a license to display the preprint in perpetuity.
It is made available under a CC-BY 4.0 International license .

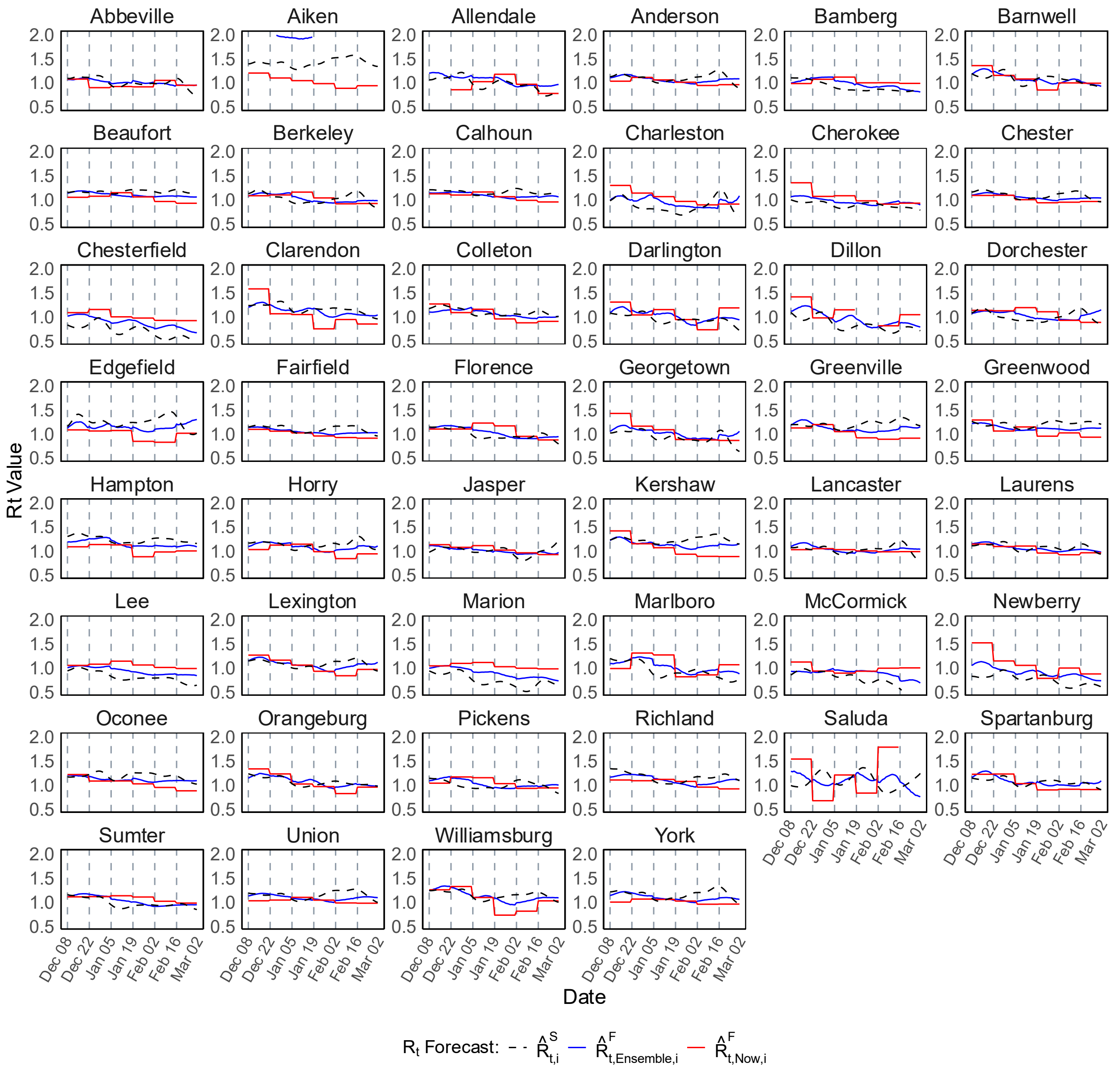

Figure S5. Forecast of R_t at the county level in SC during Scenario-2 (December 11, 2022 – March 04, 2023). This figure presents the forecast of R_t for all 46 counties in SC. The plots compare the ensemble-based forecast (blue lines) and forecasts generated using the EpiNow2 R package (red lines) alongside the spatially (covariate-adjusted) smoothed estimates ($\widehat{R}^{S}_{t,Now,i}$, black dashed lines), where i represents the county, t denotes the time point (day), and "Now" refers to the EpiNow2 method. The forecasts were generated for 14-day ahead predictions over 84 days period using a rolling window approach.

medRxiv preprint doi: https://doi.org/10.1101/2025.06.24.25330210; this version posted June 24, 2025. The copyright holder for this preprint
(which was not certified by peer review) is the author/funder, who has granted medRxiv a license to display the preprint in perpetuity.
It is made available under a CC-BY 4.0 International license .

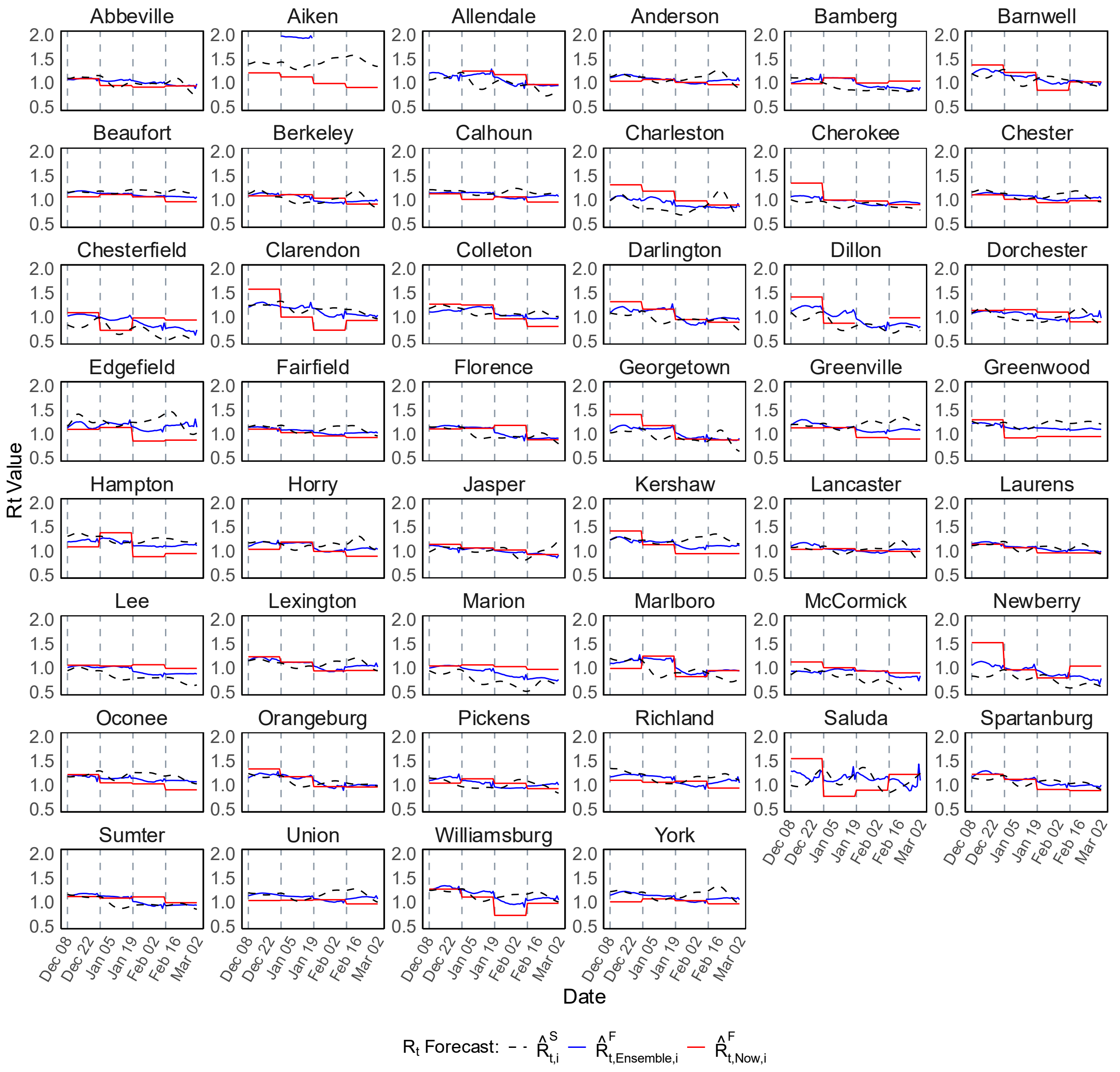

Figure S6. Forecast of R_t at the county level in SC during Scenario-2 (December 11, 2022 – March 04, 2023). This figure presents the forecast of R_t for all 46 counties in SC. The plots compare the ensemble-based forecast (blue lines) and forecasts generated using the EpiNow2 R package (red lines) alongside the spatially (covariate-adjusted) smoothed estimates ($\widehat{R}^{S}_{t,Now,i}$, black dashed lines), where i represents the county, t denotes the time point (day), and "Now" refers to the EpiNow2 method. The forecasts were generated for 21-day ahead predictions over 84 days period using a rolling window approach.

medRxiv preprint doi: https://doi.org/10.1101/2025.06.24.25330210; this version posted June 24, 2025. The copyright holder for this preprint (which was not certified by peer review) is the author/funder, who has granted medRxiv a license to display the preprint in perpetuity.
It is made available under a CC-BY 4.0 International license .

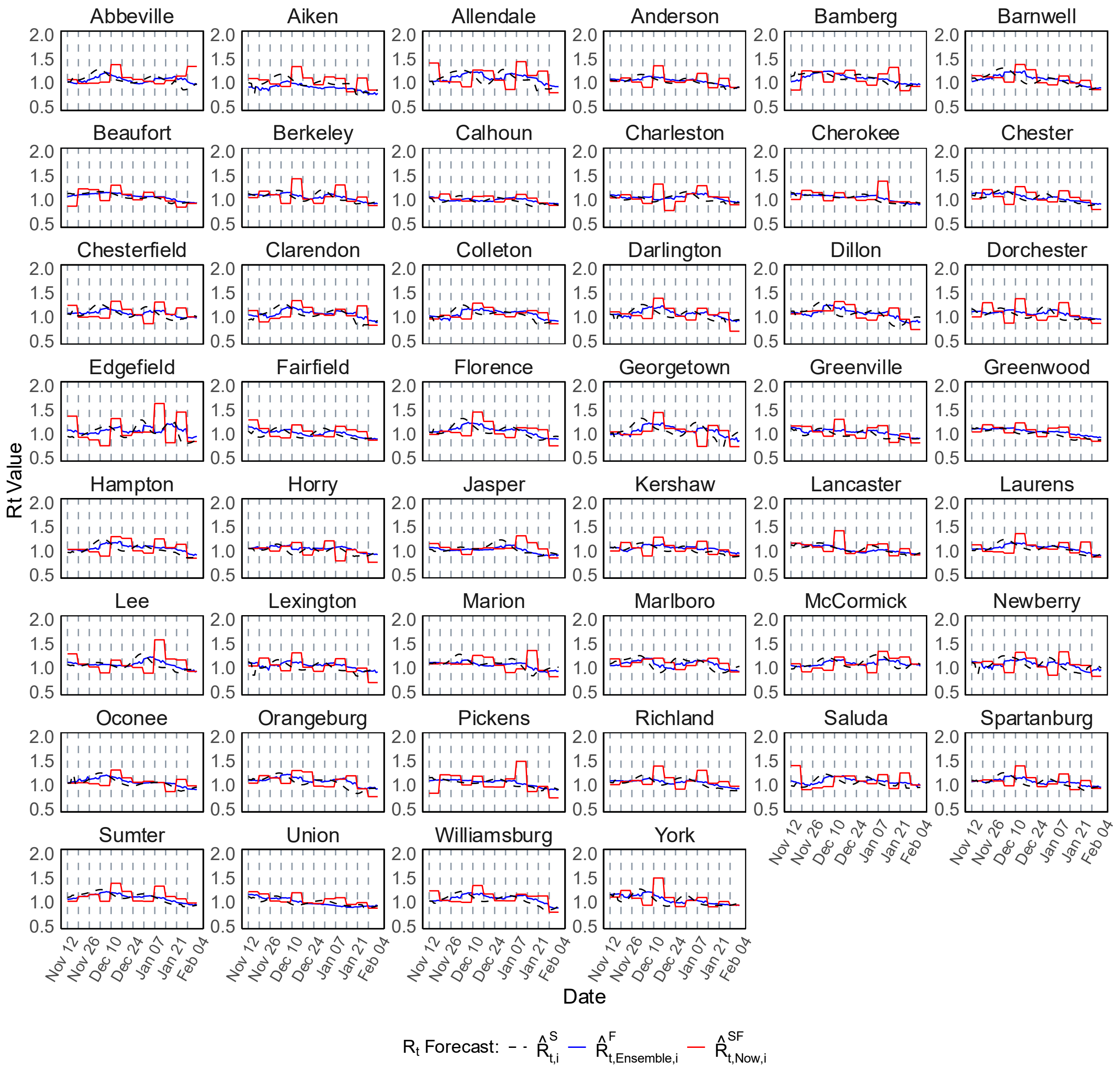

Figure S7. Forecast of R_t at the county level in SC during Scenario-1 (November 11, 2020 – February 02, 2021). This figure presents the forecast of R_t for all 46 counties in SC. The plots compare the ensemble-based forecast (blue lines) and forecasts generated using the EpiNow2 R package and spatially (covariate-adjusted) smoothed ($\widehat{R}^{SF}_{t,Now,i}$, red lines) alongside the spatially (covariate-adjusted) smoothed estimates ($\widehat{R}^{S}_{t,i}$, black dashed lines), where i represents the county, t denotes the time point (day), and "Now" refers to the EpiNow2 method. The forecasts were generated for 7-day ahead predictions over 84 days period using a rolling window approach.

medRxiv preprint doi: https://doi.org/10.1101/2025.06.24.25330210; this version posted June 24, 2025. The copyright holder for this preprint (which was not certified by peer review) is the author/funder, who has granted medRxiv a license to display the preprint in perpetuity.
It is made available under a CC-BY 4.0 International license .

Table S2. Forecast accuracy metrics for the effective reproductive number (R_t) in SC counties during two forecast periods: November 11, 2020 – February 02, 2021 (Scenario-1) and December 11, 2022 – March 04, 2023 (Scenario-2). Here forecast accuracy was calculated comparing the R_t forecast with the initial estimates of R_t. Forecast models were trained with data from June 01, 2020, to November 10, 2020, for Scenario-1, and from May 01, 2022, to December 10, 2022, for Scenario-2. The forecasting approach employed a rolling window method for 7-day, 14-day, and 21-day ahead predictions over 84 days period. The table presents the median percentage agreement (PA) across counties along with the interquartile range (IQR) of county PAs.

Forecast Method	Effective Reproductive Number Forecast: Percentage Agreement (PA), Median (IQR)					
	Forecast Period: November 11, 2020 – February 02, 2021 (Scenario-1)			Forecast Period: December 11, 2022 – March 04, 2023 (Scenario-2)		
	7-day ahead	14-day ahead	21-day ahead	7-day ahead	14-day ahead	21-day ahead
EpiNow2	85.7% (82.0% – 90.8%)	84.6% (80.4% – 90.2%)	87.2% (82.9% – 90.8%)	88.6% (86.9% – 90.5%)	88.5% (83.2% – 91.6%)	88.3% (83.2% – 92.1%)
Ensemble	93.5% (90.9% – 95.3%)	92.1% (89.9% – 94.6%)	91.9% (89.0% – 93.9%)	89.8% (87.9% – 93.2%)	89.4% (87.9% – 93.0%)	89.4% (87.6% – 92.9%)

medRxiv preprint doi: https://doi.org/10.1101/2025.06.24.25330210; this version posted June 24, 2025. The copyright holder for this preprint (which was not certified by peer review) is the author/funder, who has granted medRxiv a license to display the preprint in perpetuity.
It is made available under a CC-BY 4.0 International license .

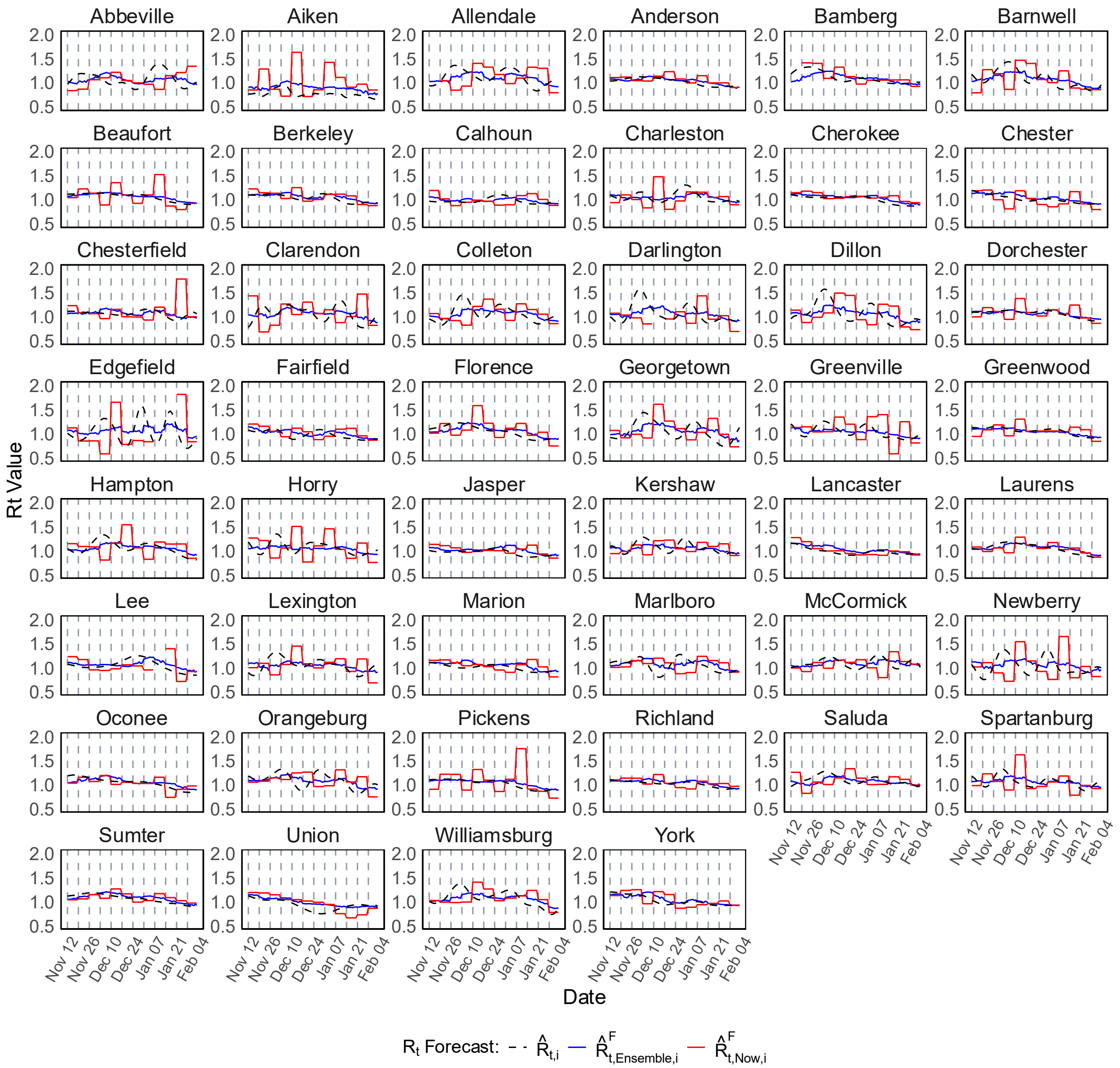

Figure S8. Forecast of R_t at the county level in SC during Scenario-1 (November 11, 2020 – February 02, 2021). This figure presents the forecast of R_t for all 46 counties in SC. The plots compare the ensemble-based forecast (blue lines), and forecasts generated using the EpiNow2 R package (red lines) alongside the initial estimates ($\hat{R}_{t,i}$, black dashed lines), where i represents the county, t denotes the time point (day), and "Now" refers to the EpiNow2 method. The forecasts were generated for 7-day ahead predictions over 84 days period using a rolling window approach.

medRxiv preprint doi: https://doi.org/10.1101/2025.06.24.25330210; this version posted June 24, 2025. The copyright holder for this preprint (which was not certified by peer review) is the author/funder, who has granted medRxiv a license to display the preprint in perpetuity.
It is made available under a CC-BY 4.0 International license .

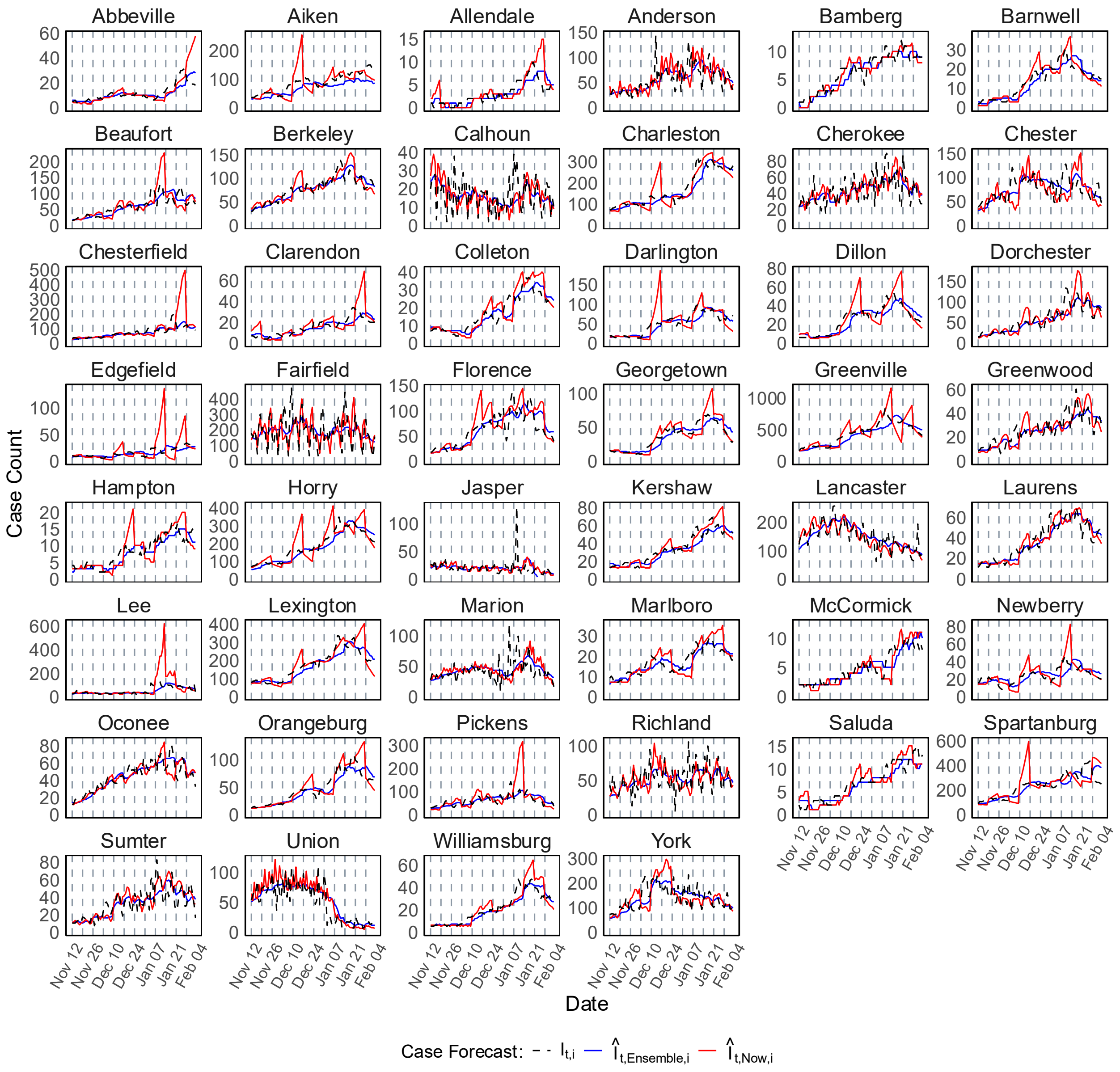

Figure S9. Forecast of COVID-19 case counts at the county level in SC during Scenario-1 (November 11, 2020 – February 02, 2021). This figure presents the forecast for COVID-19 case counts for all 46 counties in SC. The plots compare EpiNow2 forecasts ($\hat{I}_{t,Now,i}$, red lines) and ensemble-based forecasts ($\hat{I}_{t,Ensemble,i}$, blue lines) against the observed daily case counts ($I_{t,i}$,black dashed lines). The forecasts were generated for 7-day ahead predictions over 84 days period using a rolling window approach.

medRxiv preprint doi: https://doi.org/10.1101/2025.06.24.25330210; this version posted June 24, 2025. The copyright holder for this preprint (which was not certified by peer review) is the author/funder, who has granted medRxiv a license to display the preprint in perpetuity.
It is made available under a CC-BY 4.0 International license .

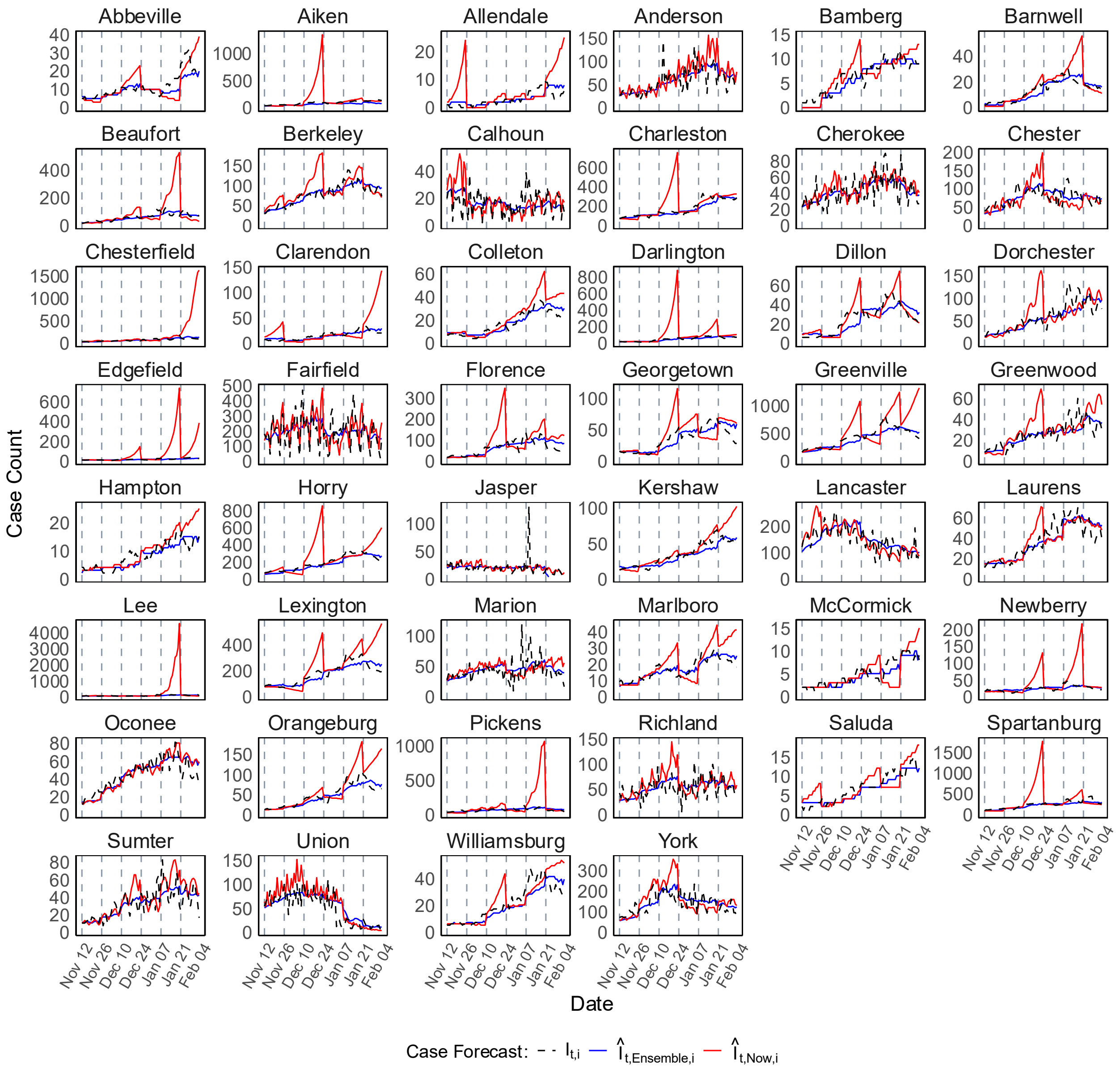

Figure S10. Forecast of COVID-19 case counts at the county level in SC during Scenario-1 (November 11, 2020 – February 02, 2021). This figure presents the forecast for COVID-19 case counts for all 46 counties in SC. The plots compare EpiNow2 forecasts ($\hat{I}_{t,Now,i}$, red lines) and ensemble-based forecasts ($\hat{I}_{t,Ensemble,i}$, blue lines) against the observed daily case counts ($I_{t,i}$,black dashed lines). The forecasts were generated for 14-day ahead predictions over 84 days period using a rolling window approach.

medRxiv preprint doi: https://doi.org/10.1101/2025.06.24.25330210; this version posted June 24, 2025. The copyright holder for this preprint (which was not certified by peer review) is the author/funder, who has granted medRxiv a license to display the preprint in perpetuity.
It is made available under a CC-BY 4.0 International license .

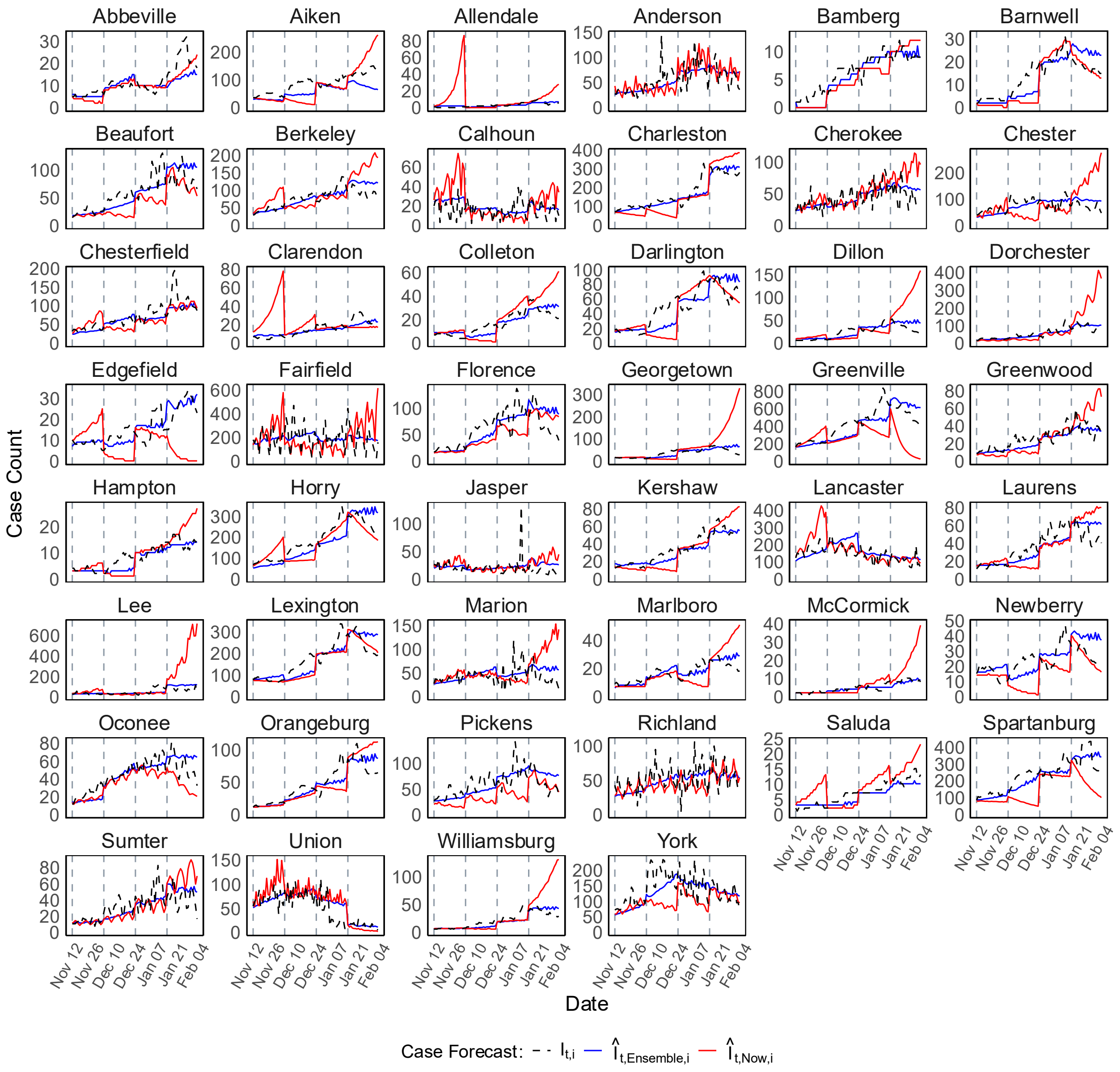

Figure S11. Forecast of COVID-19 case counts at the county level in SC during Scenario-1 (November 11, 2020 – February 02, 2021). This figure presents the forecast for COVID-19 case counts for all 46 counties in SC. The plots compare EpiNow2 forecasts ($\hat{I}_{t,Now,i}$, red lines) and ensemble-based forecasts ($\hat{I}_{t,Ensemble,i}$, blue lines) against the observed daily case counts ($I_{t,i}$,black dashed lines). The forecasts were generated for 21-day ahead predictions over 84 days period using a rolling window approach.

medRxiv preprint doi: https://doi.org/10.1101/2025.06.24.25330210; this version posted June 24, 2025. The copyright holder for this preprint (which was not certified by peer review) is the author/funder, who has granted medRxiv a license to display the preprint in perpetuity.
It is made available under a CC-BY 4.0 International license .

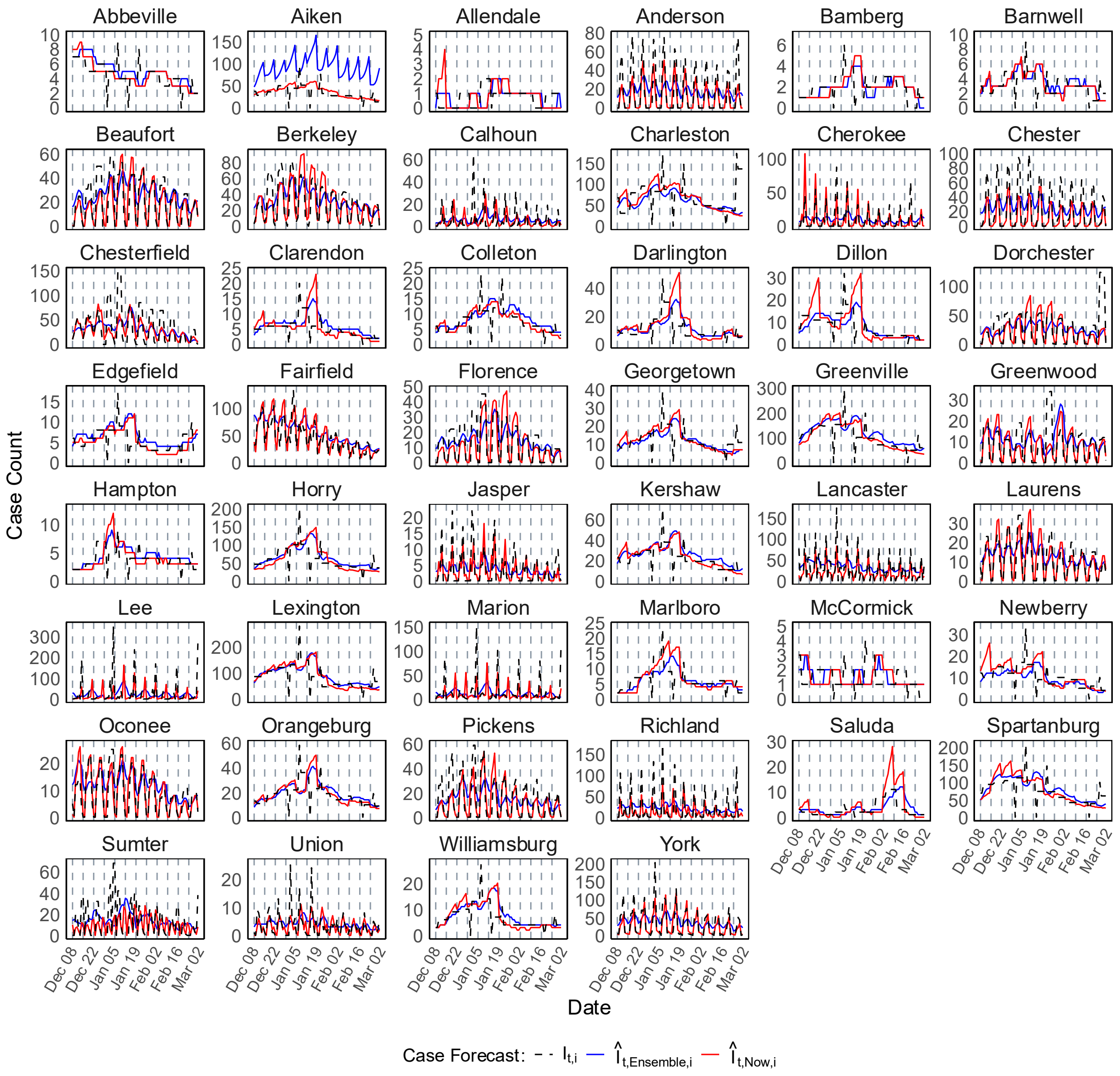

Figure S12. Forecast of COVID-19 case counts at the county level in SC during Scenario-2 (December 11, 2022 – March 04, 2023). This figure presents the forecast for COVID-19 case counts for all 46 counties in SC. The plots compare EpiNow2 forecasts ($\hat{I}_{t,Now,i}$, red lines) and ensemble-based forecasts ($\hat{I}_{t,Ensemble,i}$, blue lines) against the observed daily case counts ($I_{t,i}$,black dashed lines). The forecasts were generated for 7-day ahead predictions over 84 days period using a rolling window approach.

medRxiv preprint doi: https://doi.org/10.1101/2025.06.24.25330210; this version posted June 24, 2025. The copyright holder for this preprint (which was not certified by peer review) is the author/funder, who has granted medRxiv a license to display the preprint in perpetuity.
It is made available under a CC-BY 4.0 International license .

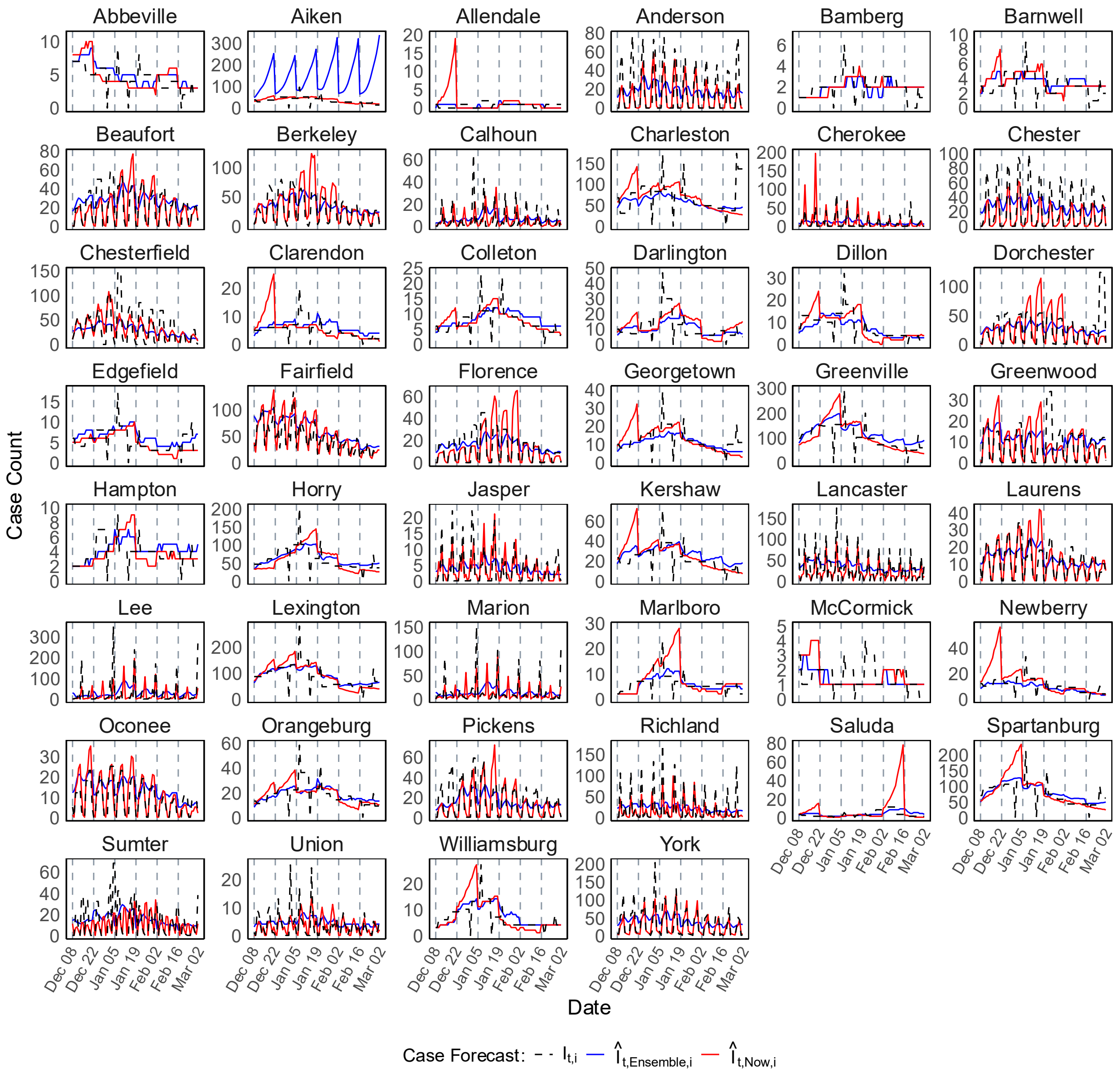

Figure S13. Forecast of COVID-19 case counts at the county level in SC during Scenario-2 (December 11, 2022 – March 04, 2023). This figure presents the forecast for COVID-19 case counts for all 46 counties in SC. The plots compare EpiNow2 forecasts ($\hat{I}_{t,Now,i}$, red lines) and ensemble-based forecasts ($\hat{I}_{t,Ensemble,i}$, blue lines) against the observed daily case counts ($I_{t,i}$,black dashed lines). The forecasts were generated for 14-day ahead predictions over 84 days period using a rolling window approach.

medRxiv preprint doi: https://doi.org/10.1101/2025.06.24.25330210; this version posted June 24, 2025. The copyright holder for this preprint (which was not certified by peer review) is the author/funder, who has granted medRxiv a license to display the preprint in perpetuity.
It is made available under a CC-BY 4.0 International license .

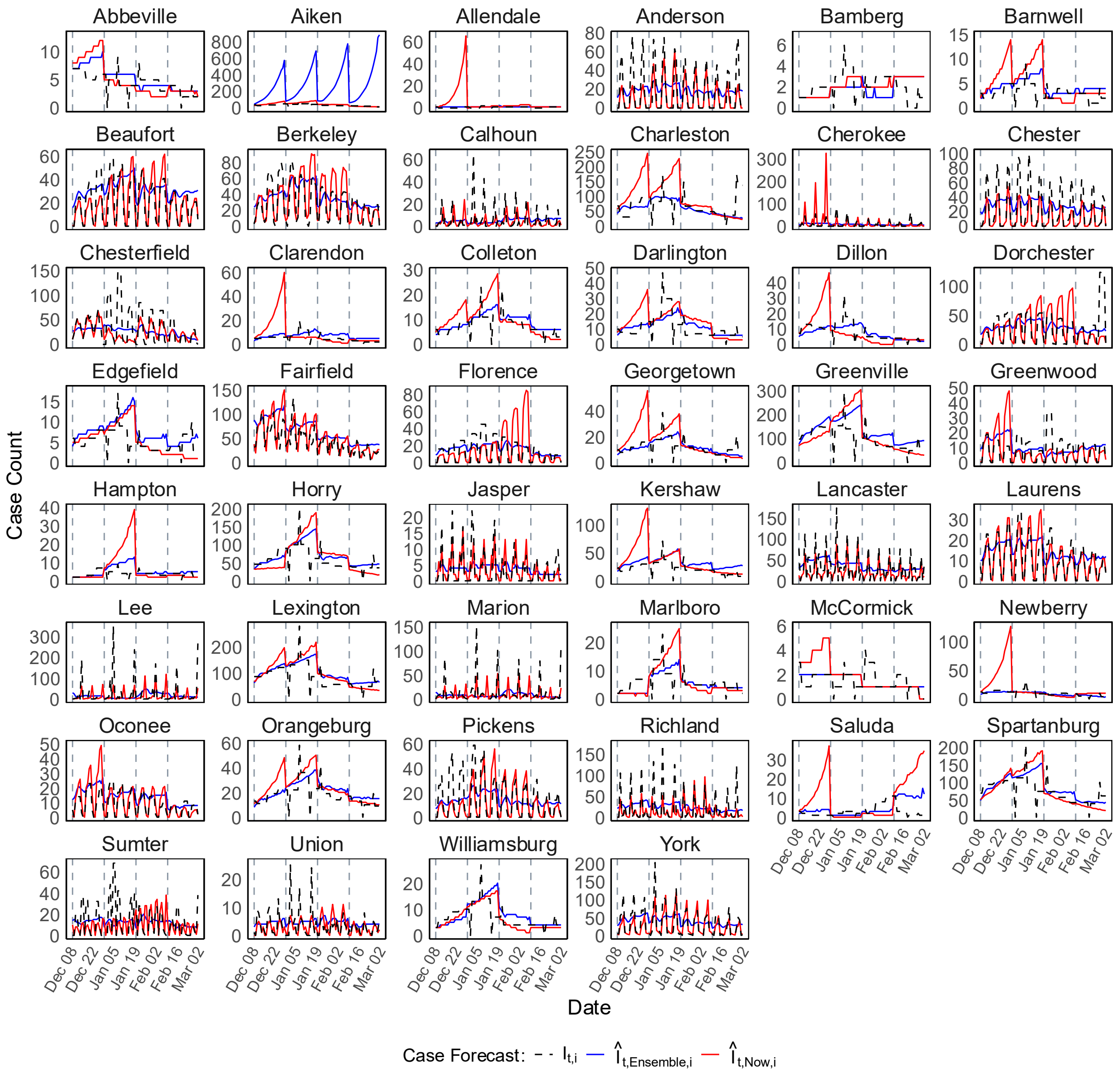

Figure S14. Forecast of COVID-19 case counts at the county level in SC during Scenario-2 (December 11, 2022 – March 04, 2023). This figure presents the forecast for COVID-19 case counts for all 46 counties in SC. The plots compare EpiNow2 forecasts ($\hat{I}_{t,Now,i}$, red lines) and ensemble-based forecasts ($\hat{I}_{t,Ensemble,i}$, blue lines) against the observed daily case counts ($I_{t,i}$,black dashed lines). The forecasts were generated for 21-day ahead predictions over 84 days period using a rolling window approach.

medRxiv preprint doi: https://doi.org/10.1101/2025.06.24.25330210; this version posted June 24, 2025. The copyright holder for this preprint (which was not certified by peer review) is the author/funder, who has granted medRxiv a license to display the preprint in perpetuity.
It is made available under a CC-BY 4.0 International license .

Table S3. Forecast accuracy metrics for individual models used in the ensemble-based forecasting of R_t at the county level in SC during Scenario-1 (November 11, 2020 – February 02, 2021) and Scenario-2 (December 11, 2022 – March 04, 2023). The models include Regression, Random Forest (RF), and XGBoost, applied to both the initial estimates, $\widehat{R}_{t,Now,i}$, and the spatially (covariate-adjusted) smoothed estimates, $\widehat{R}^{S}_{t,Now,i}$. Forecast accuracy is evaluated using percentage agreement (PA) with median and interquartile range (IQR). The forecasting approach involved 7-day, 14-day, and 21-day ahead predictions over 84 days period using a rolling window method. The accuracy metrics for each individual forecasting model are summarized in this table.

Forecast Method	Effective Reproductive Number Forecast: Percentage Agreement (PA), Median (IQR)					
	Forecast Period: November 11, 2020 – February 02, 2021			Forecast Period: December 11, 2022 – March 04, 2023		
	7-day ahead	14-day ahead	21-day ahead	7-day ahead	14-day ahead	21-day ahead
Regression	90.5% (86.8% – 92.9%)	89.0% (85.4% – 92.2%)	89.0% (86.0% – 92.4%)	86.9% (81.9% – 88.7%)	86.9% (81.9% – 88.7%)	86.0% (81.5% – 88.3%)
Regression (Smooth)	94.1% (92.9% – 94.9%)	91.0% (89.8% – 92.6%)	89.2% (87.3% – 91.0%)	90.9% (89.4% – 92.1%)	91.0% (89.4% – 92.1%)	90.6% (89.2% – 91.7%)
RF	94.1% (92.2% – 95.2%)	93.8% (92.1% – 94.9%)	92.8% (90.1% – 94.5%)	86.7% (82.5% – 91.4%)	88.4% (82.5% – 91.0%)	87.4% (83.8% – 90.5%)
RF (Smooth)	96.5% (96.0% – 97.0%)	95.3% (94.1% – 96.1%)	93.1% (92.1% – 93.7%)	96.9% (95.9% – 97.5%)	95.3% (94.0% – 96.3%)	93.4% (91.6% – 94.5%)
XGBoost	89.7% (86.3% – 92.1%)	89.7% (86.2% – 92.1%)	89.4% (85.8% – 91.7%)	86.7% (82.3% – 88.8%)	86.5% (82.1% – 88.8%)	86.8% (81.0% – 89.3%)
XGBoost (Smooth)	93.2% (92.1% – 94.2%)	90.8% (90.4% – 93.1%)	92.4% (90.9% – 93.4%)	90.5% (88.3% – 91.8%)	90.6% (88.3% – 91.9%)	90.5% (88.3% – 91.8%)

medRxiv preprint doi: https://doi.org/10.1101/2025.06.24.25330210; this version posted June 24, 2025. The copyright holder for this preprint (which was not certified by peer review) is the author/funder, who has granted medRxiv a license to display the preprint in perpetuity.
It is made available under a CC-BY 4.0 International license .

EpiNow2 (Smooth)	87.6% (86.6% – 88.9%)	86.2% (83.8% – 88.2%)	89.7% (87.5% – 92.3%)	87.2% (84.0% – 89.9%)	86.7% (84.2% – 89.9%)	87.5% (84.8% – 90.3%)

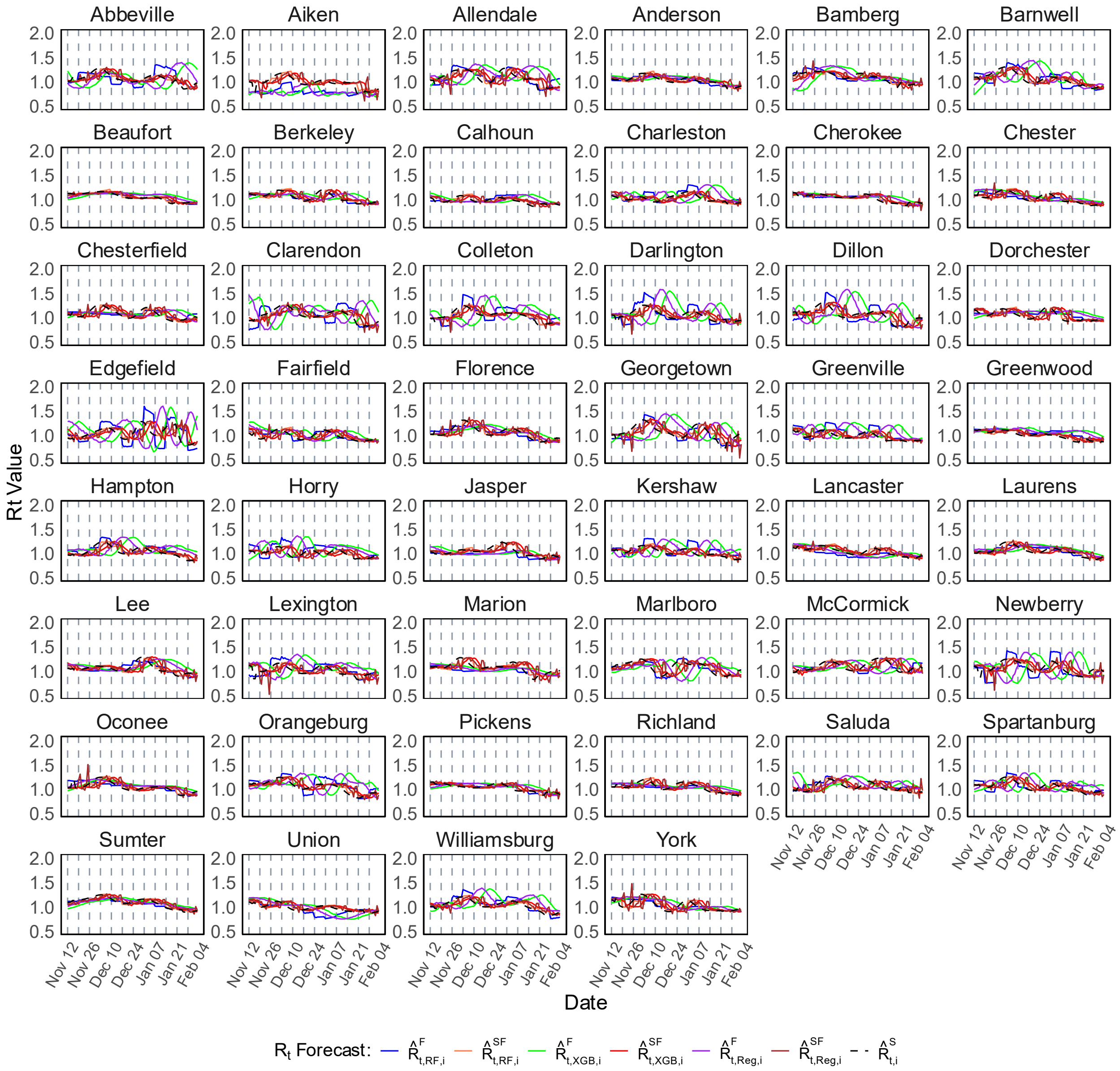

Figure S15. Forecast of the effective reproductive number (R_t) at the county level in SC during Scenario-1 (November 11, 2020 – February 02, 2021). This figure presents the forecast of R_t for all 46 counties in SC. The plots compare forecasts generated using multiple individual models, alongside the spatially (covariate-adjusted) smoothed estimates (black dashed lines, $\hat{R}^S_{t,i}$), where i presents the county, t denotes the time point (day), and "Now" refers to

medRxiv preprint doi: https://doi.org/10.1101/2025.06.24.25330210; this version posted June 24, 2025. The copyright holder for this preprint
(which was not certified by peer review) is the author/funder, who has granted medRxiv a license to display the preprint in perpetuity.
It is made available under a CC-BY 4.0 International license .

the **EpiNow2** method. The forecasts were generated for 7-day ahead predictions over 84 days period using a rolling window approach.

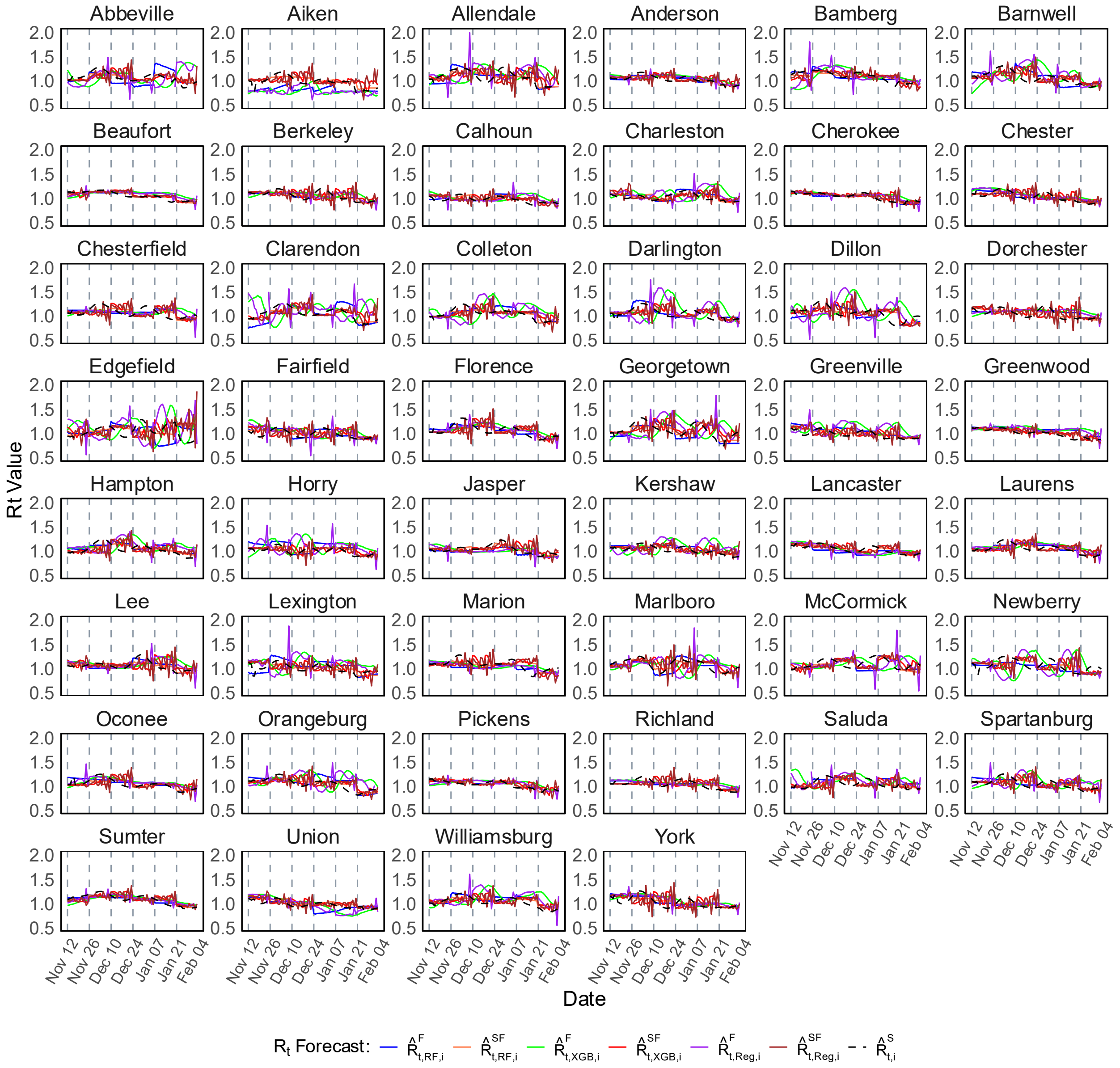

Figure S16. Forecast of the effective reproductive number (R_t) at the county level in SC during Scenario-1 (November 11, 2020 – February 02, 2021). This figure presents the forecast of R_t for all 46 counties in SC. The plots compare forecasts generated using multiple individual models, alongside the spatially (covariate-adjusted) smoothed estimates (black dashed lines, $\hat{R}^{S}_{t,i}$), where i presents the county, t denotes the time point (day), and "Now" refers to the **EpiNow2** method. The forecasts were generated for 14-day ahead predictions over 84 days period using a rolling window approach.

medRxiv preprint doi: https://doi.org/10.1101/2025.06.24.25330210; this version posted June 24, 2025. The copyright holder for this preprint (which was not certified by peer review) is the author/funder, who has granted medRxiv a license to display the preprint in perpetuity.
It is made available under a CC-BY 4.0 International license .

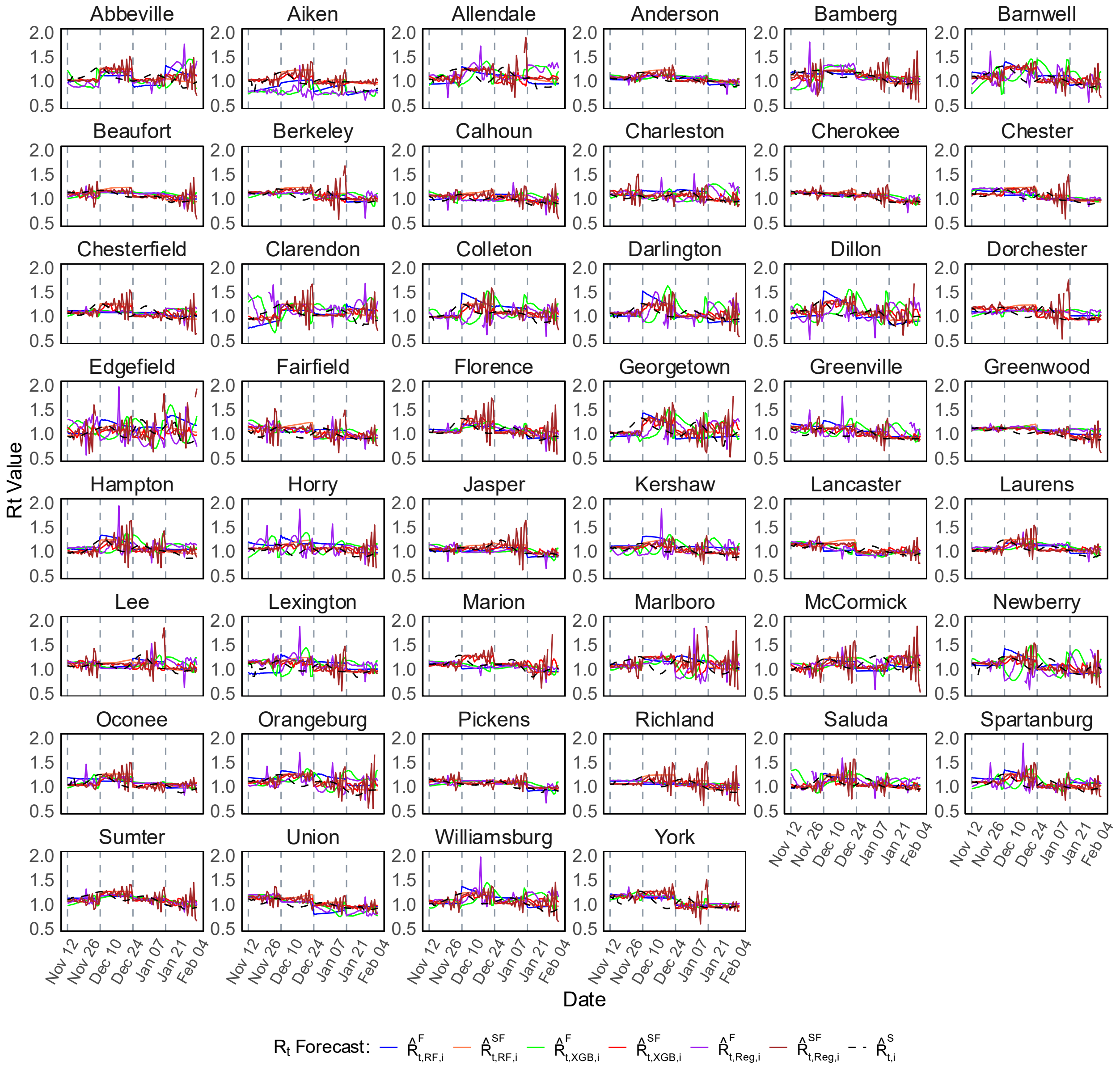

Figure S17. Forecast of the effective reproductive number (R_t) at the county level in SC during Scenario-1 (November 11, 2020 – February 02, 2021). This figure presents the forecast of R_t for all 46 counties in SC. The plots compare forecasts generated using multiple individual models, alongside the spatially (covariate-adjusted) smoothed estimates (black dashed lines, $\hat{R}^{S}_{t,i}$), where i presents the county, t denotes the time point (day), and "Now" refers to the **EpiNow2** method. The forecasts were generated for 21-day ahead predictions over 84 days period using a rolling window approach.

medRxiv preprint doi: https://doi.org/10.1101/2025.06.24.25330210; this version posted June 24, 2025. The copyright holder for this preprint (which was not certified by peer review) is the author/funder, who has granted medRxiv a license to display the preprint in perpetuity.
It is made available under a CC-BY 4.0 International license .

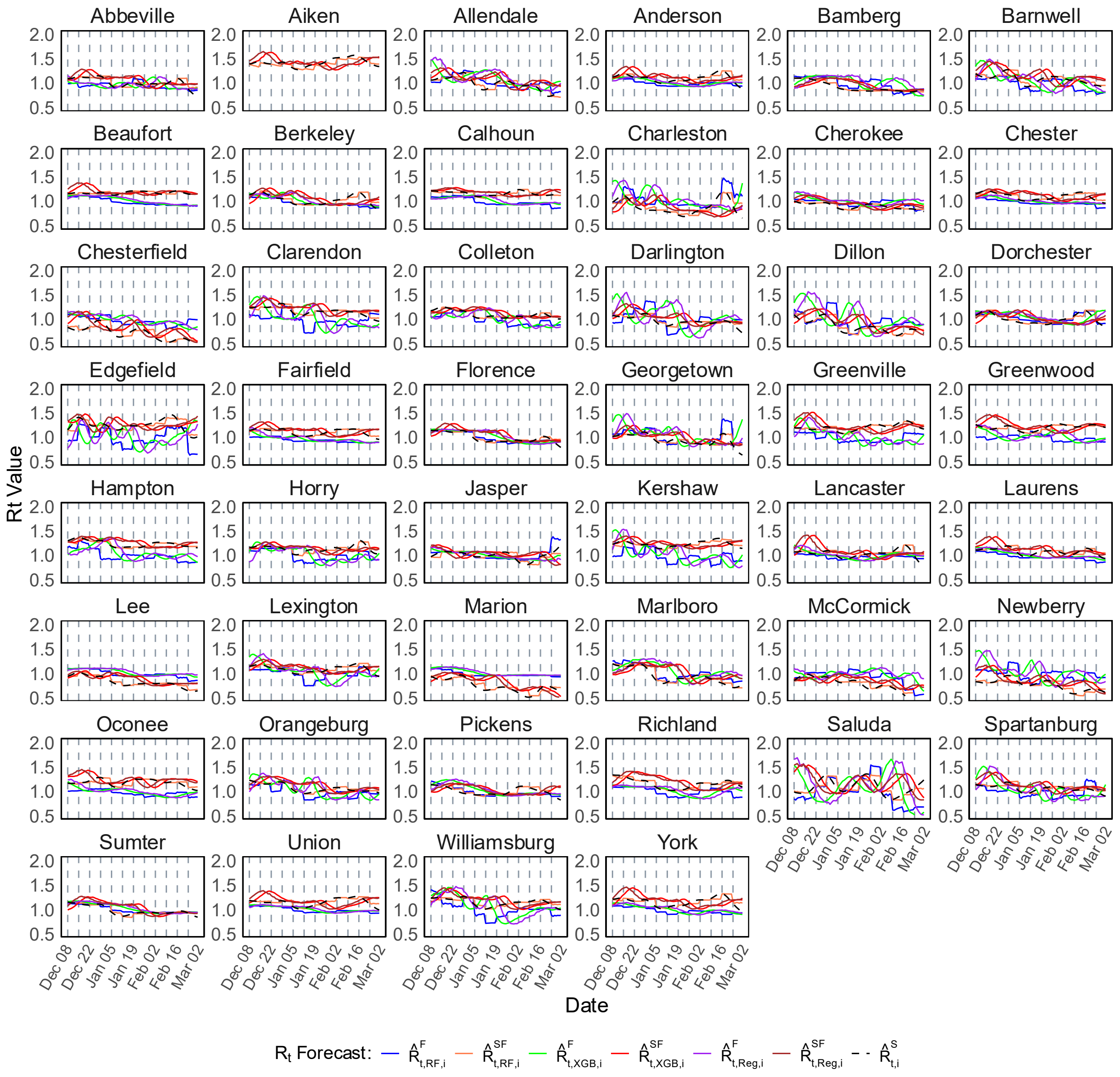

Figure S18. Forecast of the effective reproductive number (R_t) at the county level in SC during Scenario-2 (December 11, 2022 – March 04, 2023). This figure presents the forecast of R_t for all 46 counties in SC. The plots compare forecasts generated using multiple individual models, alongside the spatially (covariate-adjusted) smoothed estimates (black dashed lines, $\hat{R}^S_{t,i}$), where i presents the county, t denotes the time point (day), and "Now" refers to the **EpiNow2** method. The forecasts were generated for 7-day ahead predictions over 84 days period using a rolling window approach.

medRxiv preprint doi: https://doi.org/10.1101/2025.06.24.25330210; this version posted June 24, 2025. The copyright holder for this preprint (which was not certified by peer review) is the author/funder, who has granted medRxiv a license to display the preprint in perpetuity.
It is made available under a CC-BY 4.0 International license .

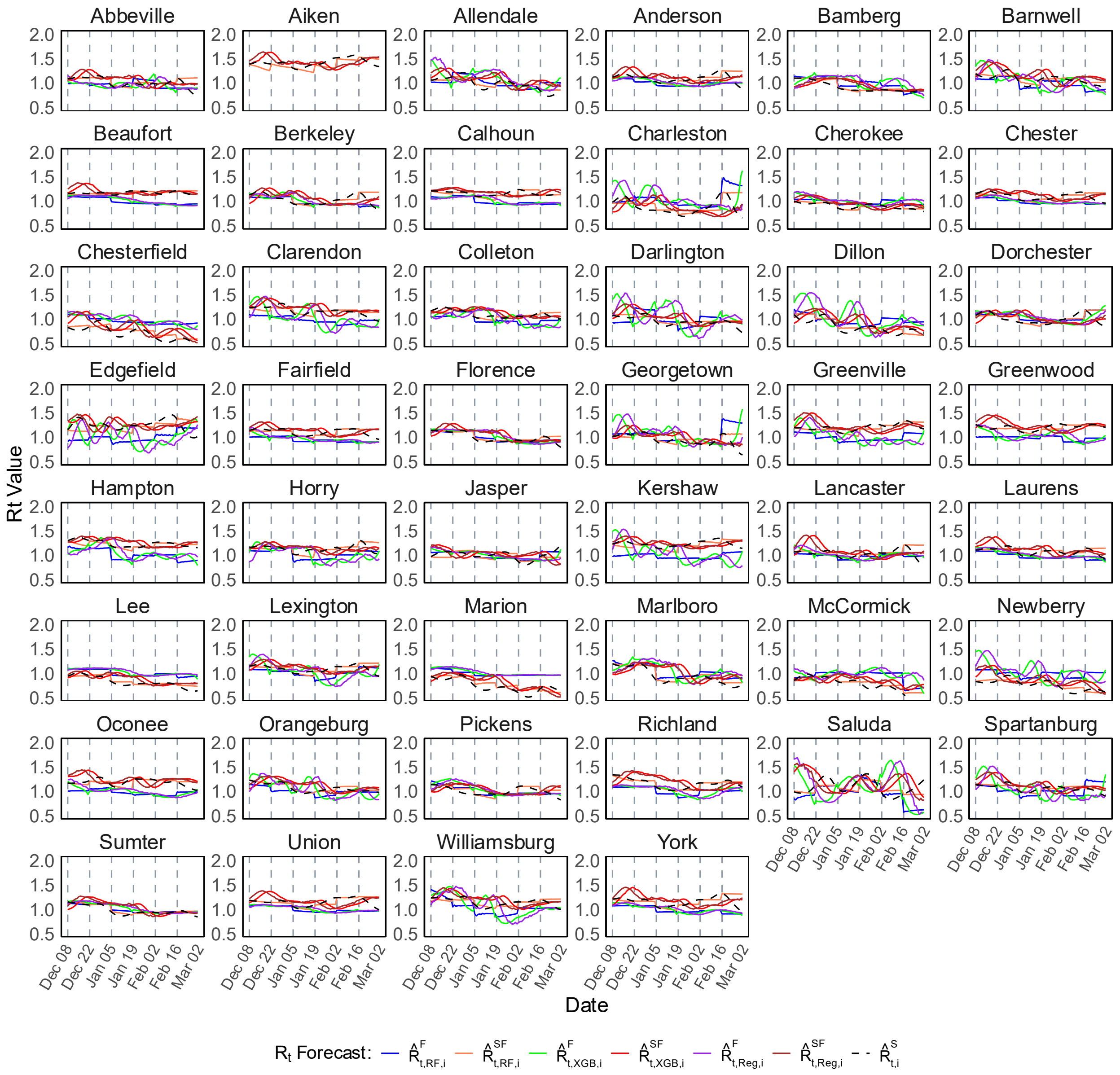

Figure S19. Forecast of the effective reproductive number (R_t) at the county level in SC during Scenario-2 (December 11, 2022 – March 04, 2023). This figure presents the forecast of R_t for all 46 counties in SC. The plots compare forecasts generated using multiple individual models, alongside the spatially (covariate-adjusted) smoothed estimates (black dashed lines, $\hat{R}^{S}_{t,i}$), where i presents the county, t denotes the time point (day), and "Now" refers to the **EpiNow2** method. The forecasts were generated for 14-day ahead predictions over 84 days period using a rolling window approach.

medRxiv preprint doi: https://doi.org/10.1101/2025.06.24.25330210; this version posted June 24, 2025. The copyright holder for this preprint (which was not certified by peer review) is the author/funder, who has granted medRxiv a license to display the preprint in perpetuity.
It is made available under a CC-BY 4.0 International license .

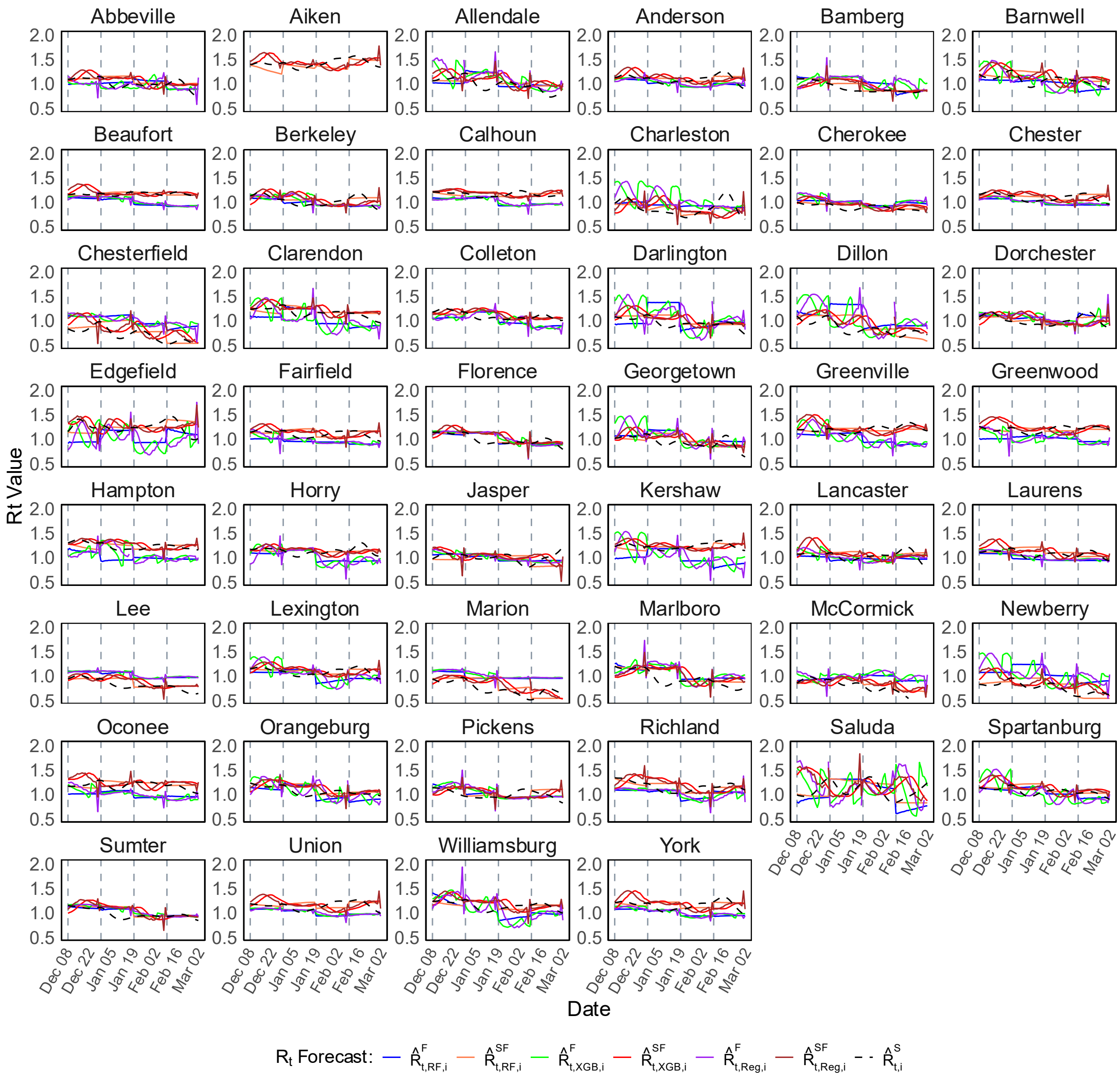

Figure S20. Forecast of the effective reproductive number (R_t) at the county level in SC during Scenario-2 (December 11, 2022 – March 04, 2023). This figure presents the forecast of R_t for all 46 counties in SC. The plots compare forecasts generated using multiple individual models, alongside the spatially (covariate-adjusted) smoothed estimates (black dashed lines, $\hat{R}^S_{t,i}$), where i presents the county, t denotes the time point (day), and "Now" refers to the **EpiNow2** method. The forecasts were generated for 21-day ahead predictions over 84 days period using a rolling window approach.

medRxiv preprint doi: https://doi.org/10.1101/2025.06.24.25330210; this version posted June 24, 2025. The copyright holder for this preprint (which was not certified by peer review) is the author/funder, who has granted medRxiv a license to display the preprint in perpetuity.
It is made available under a CC-BY 4.0 International license .

Table S4. Forecast accuracy metrics for COVID-19 daily case counts forecasts using individual models at the county level in SC during Scenario-1 (November 11, 2020 – February 02, 2021) and Scenario-2 (December 11, 2022 – March 04, 2023). The last row presents accuracy of R_t forecasting using EpiNow2, followed by spatial (covariate-adjusted) smoothing. We employed a probabilistic approach using a Poisson model to generate case forecasts based on the forcasted R_t values, including $\hat{R}^{F}_{t,Reg,i}$, $\hat{R}^{F}_{t,RF,i}$, $\hat{R}^{F}_{t,XGB,i}$, $\hat{R}^{SF}_{t,Reg,i}$, $\hat{R}^{SF}_{t,RF,i}$, and $\hat{R}^{SF}_{t,XGB,i}$, then evaluated by comparing with observed case counts.. Accuracy was measured using percentage agreement (PA) with median and interquartile range (IQR). The forecasts were generated for 7-day, 14-day, and 21-day ahead predictions over 84 days period using a rolling window approach.

Forecast Method	COVID-19 Daily Cases Forecast: Percentage Agreement (PA), Median (IQR)					
	Forecast Period: November 11, 2020 – February 02, 2021			Forecast Period: December 11, 2022 – March 04, 2023		
	7-day ahead	14-day ahead	21-day ahead	7-day ahead	14-day ahead	21-day ahead
Regression	85.9% (83.6% – 87.9%)	84.6% (82.7% – 87.7%)	79.0% (73.6% – 81.2%)	78.3% (72.3% – 81.2%)	78.0% (72.0% – 81.6%)	77.1% (71.9% – 81.5%)
Regression (Smooth)	85.9% (84.1% – 88.4%)	83.0% (80.3% – 85.3%)	79.6% (77.8% – 82.6%)	74.9% (71.0% – 79.2%)	71.7% (66.8% – 76.9%)	69.4% (61.7% – 74.4%)
RF	85.3% (83.4% – 88.2%)	84.4% (82.5% – 87.7%)	83.1% (80.3% – 85.6%)	81.3% (76.7% – 83.2%)	82.7% (79.9% – 85.3%)	77.1% (71.9% – 81.5%)
RF (Smooth)	86.0% (82.8% – 88.1%)	84.0% (80.5% – 86.4%)	80.6% (78.6% – 84.2%)	76.0% (72.5% – 79.5%)	72.7% (67.8% – 77.0%)	68.7% (61.2% – 76.3%)
XGBoost	82.8% (78.6% – 85.8%)	80.5% (76.3% – 83.9%)	76.3% (72.1% – 80.0%)	80.4% (75.6% – 82.1%)	80.4% (77.2% – 84.0%)	78.4% (71.9% – 81.9%)

medRxiv preprint doi: https://doi.org/10.1101/2025.06.24.25330210; this version posted June 24, 2025. The copyright holder for this preprint (which was not certified by peer review) is the author/funder, who has granted medRxiv a license to display the preprint in perpetuity.
It is made available under a CC-BY 4.0 International license .

XGBoost (Smooth)	86.5% (84.4% – 88.6%)	83.4% (80.4% – 85.3%)	78.7% (76.1% – 80.8%)	76.0% (72.3% – 78.5%)	72.7% (67.7% – 76.7%)	69.7% (60.3% – 75.6%)

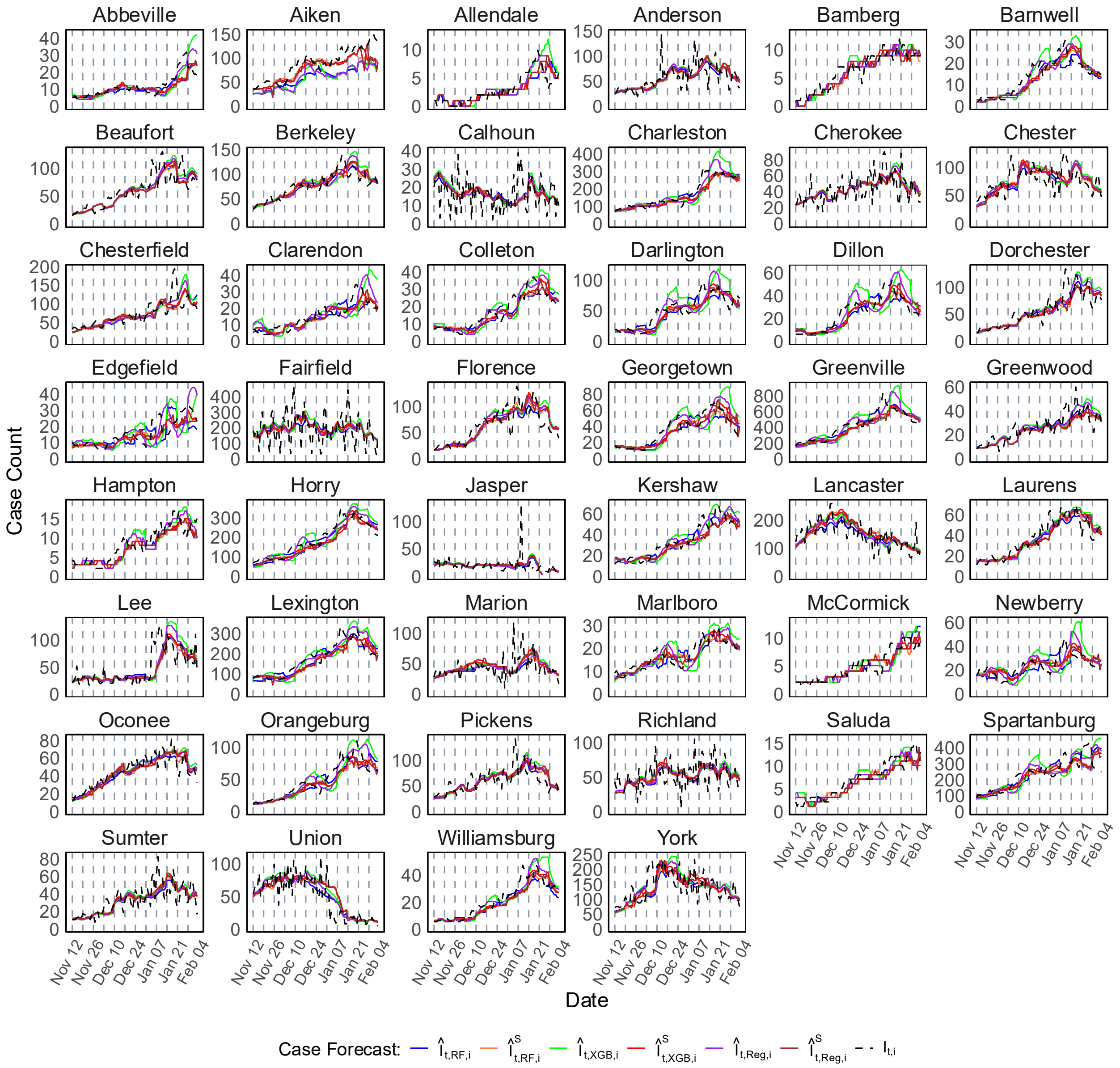

Figure S21. Forecast of COVID-19 case counts at the county level in SC during Scenario-1 (November 11, 2020 – February 02, 2021). This figure presents the forecast of COVID-19 case counts for all 46 counties in SC. The plots compare forecasts generated using multiple individual models against the observed daily case counts (black dashed

medRxiv preprint doi: https://doi.org/10.1101/2025.06.24.25330210; this version posted June 24, 2025. The copyright holder for this preprint (which was not certified by peer review) is the author/funder, who has granted medRxiv a license to display the preprint in perpetuity.
It is made available under a CC-BY 4.0 International license .

lines, $I_{t,i}$), where i represents the county and t denotes the time point (day). The forecasts were generated for 7-day ahead predictions over 84 days period using a rolling window approach.

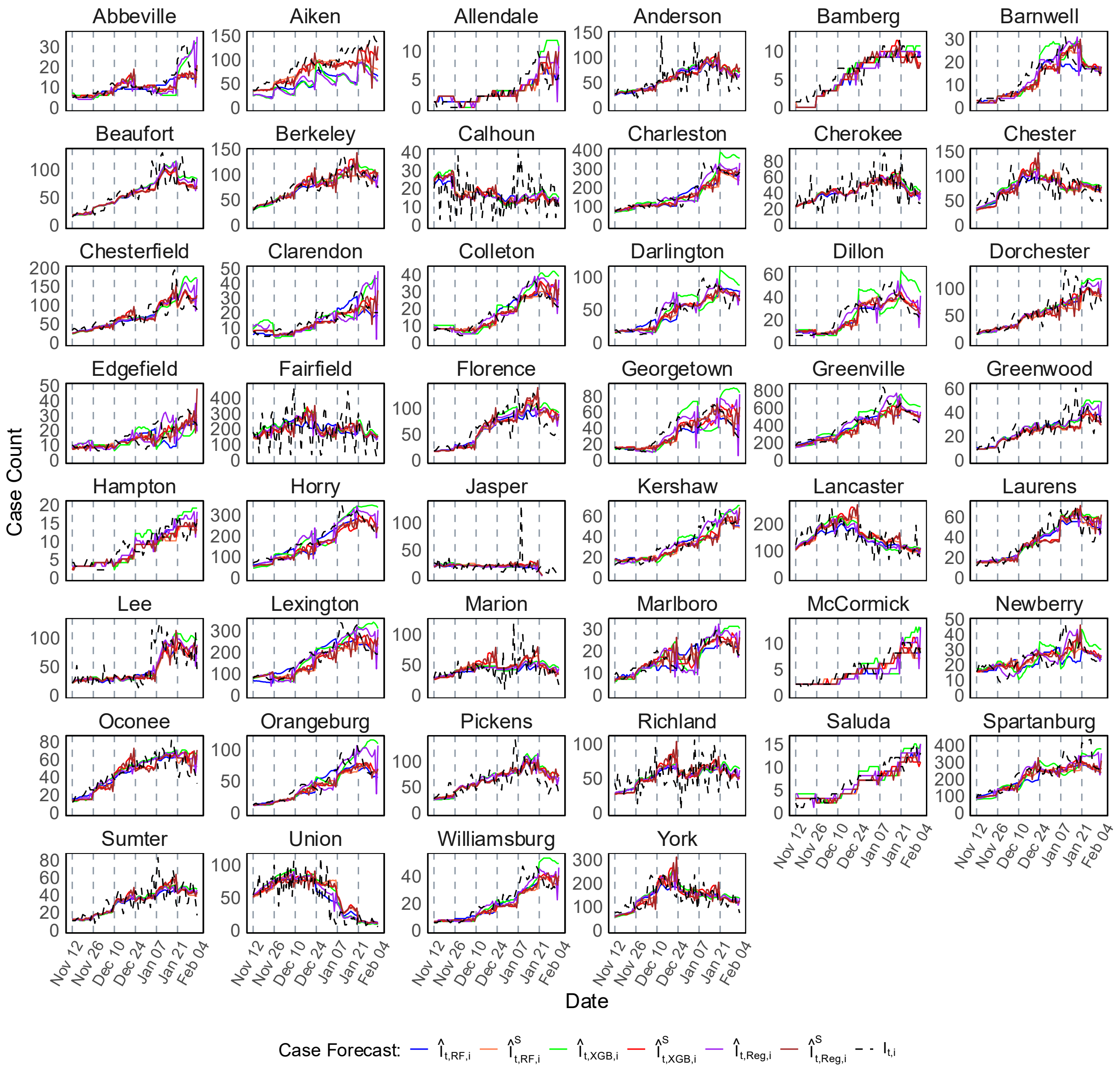

Figure S22. Forecast of COVID-19 case counts at the county level in SC during Scenario-1 (November 11, 2020 – February 02, 2021). This figure presents the forecast of COVID-19 case counts for all 46 counties in SC. The plots compare forecasts generated using multiple individual models against the observed daily case counts (black dashed lines, $I_{t,i}$), where i represents the county and t denotes the time point (day). The forecasts were generated for 14-day ahead predictions over 84 days period using a rolling window approach.

medRxiv preprint doi: https://doi.org/10.1101/2025.06.24.25330210; this version posted June 24, 2025. The copyright holder for this preprint (which was not certified by peer review) is the author/funder, who has granted medRxiv a license to display the preprint in perpetuity.
It is made available under a CC-BY 4.0 International license .

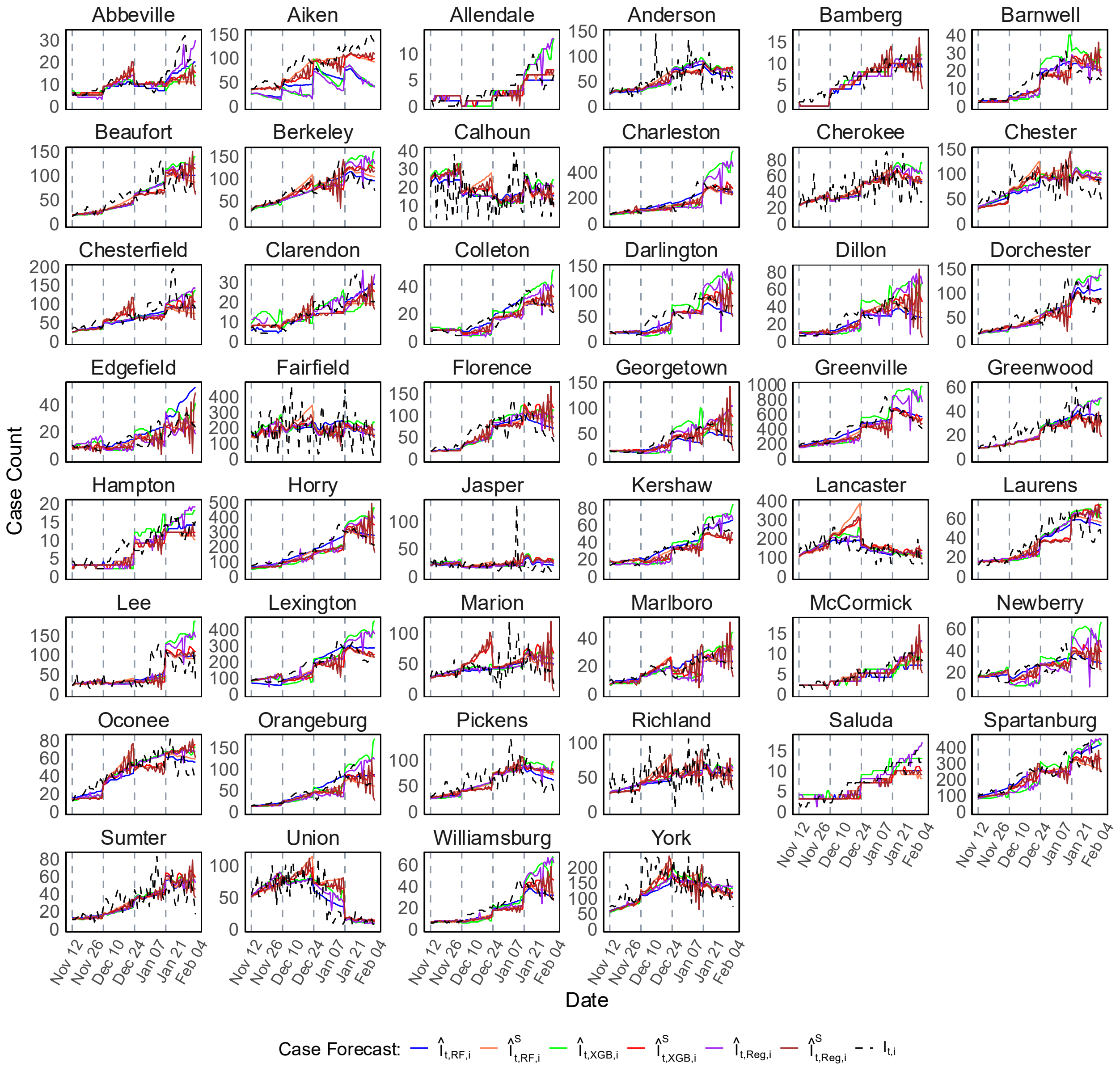

Figure S23. Forecast of COVID-19 case counts at the county level in SC during Scenario-1 (November 11, 2020 – February 02, 2021). This figure presents the forecast of COVID-19 case counts for all 46 counties in SC. The plots compare forecasts generated using multiple individual models against the observed daily case counts (black dashed lines, $I_{t,i}$), where i represents the county and t denotes the time point (day). The forecasts were generated for 21-day ahead predictions over 84 days period using a rolling window approach.

medRxiv preprint doi: https://doi.org/10.1101/2025.06.24.25330210; this version posted June 24, 2025. The copyright holder for this preprint (which was not certified by peer review) is the author/funder, who has granted medRxiv a license to display the preprint in perpetuity.
It is made available under a CC-BY 4.0 International license .

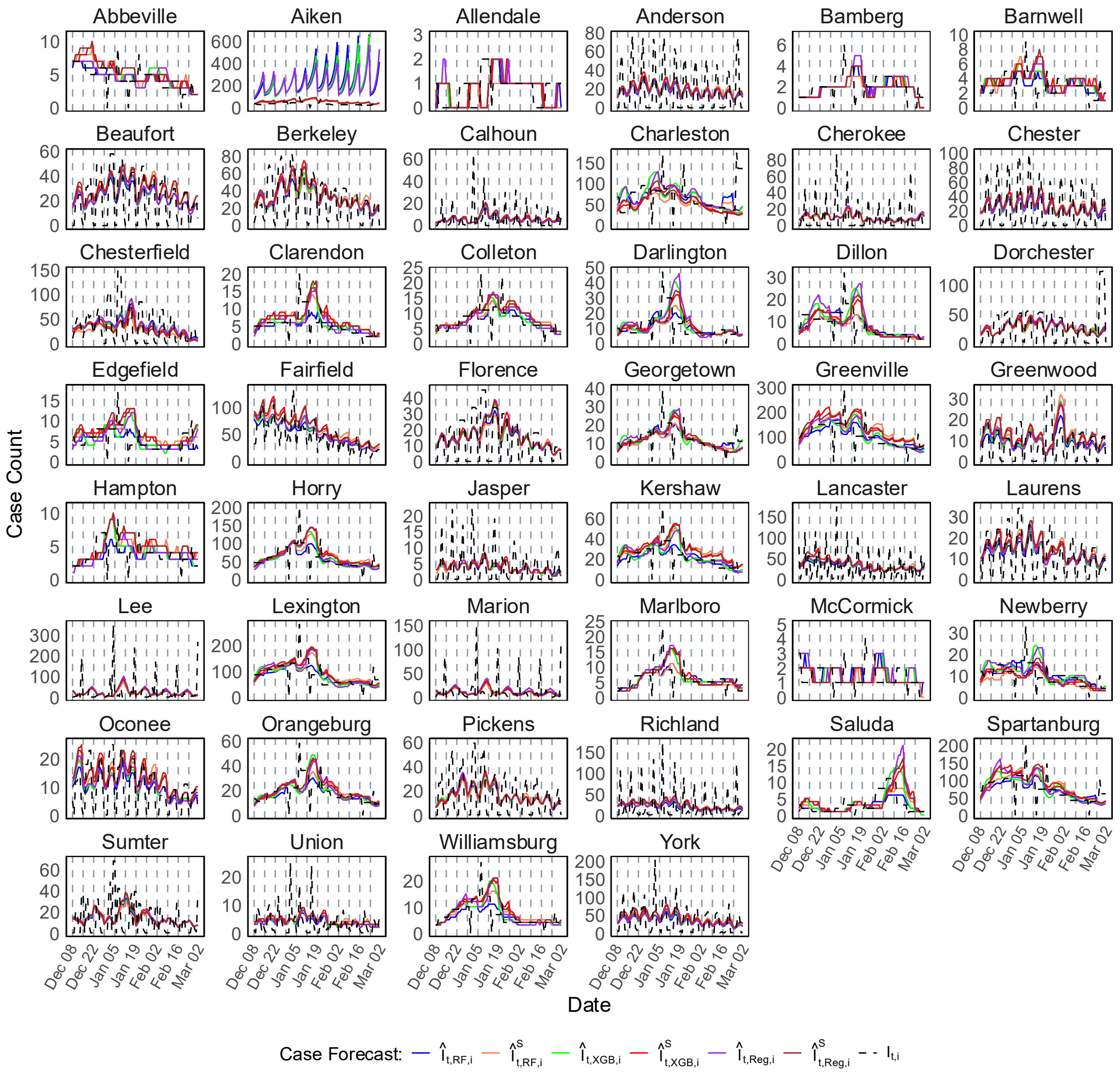

Figure S24. Forecast of COVID-19 case counts at the county level in SC during Scenario-2 (December 11, 2022 – March 04, 2023). This figure presents the forecast of COVID-19 case counts for all 46 counties in SC. The plots compare forecasts generated using multiple individual models against the observed daily case counts (black dashed lines, $I_{t,i}$), where i represents the county and t denotes the time point (day). The forecasts were generated for 7-day ahead predictions over 84 days period using a rolling window approach.

medRxiv preprint doi: https://doi.org/10.1101/2025.06.24.25330210; this version posted June 24, 2025. The copyright holder for this preprint (which was not certified by peer review) is the author/funder, who has granted medRxiv a license to display the preprint in perpetuity.
It is made available under a CC-BY 4.0 International license .

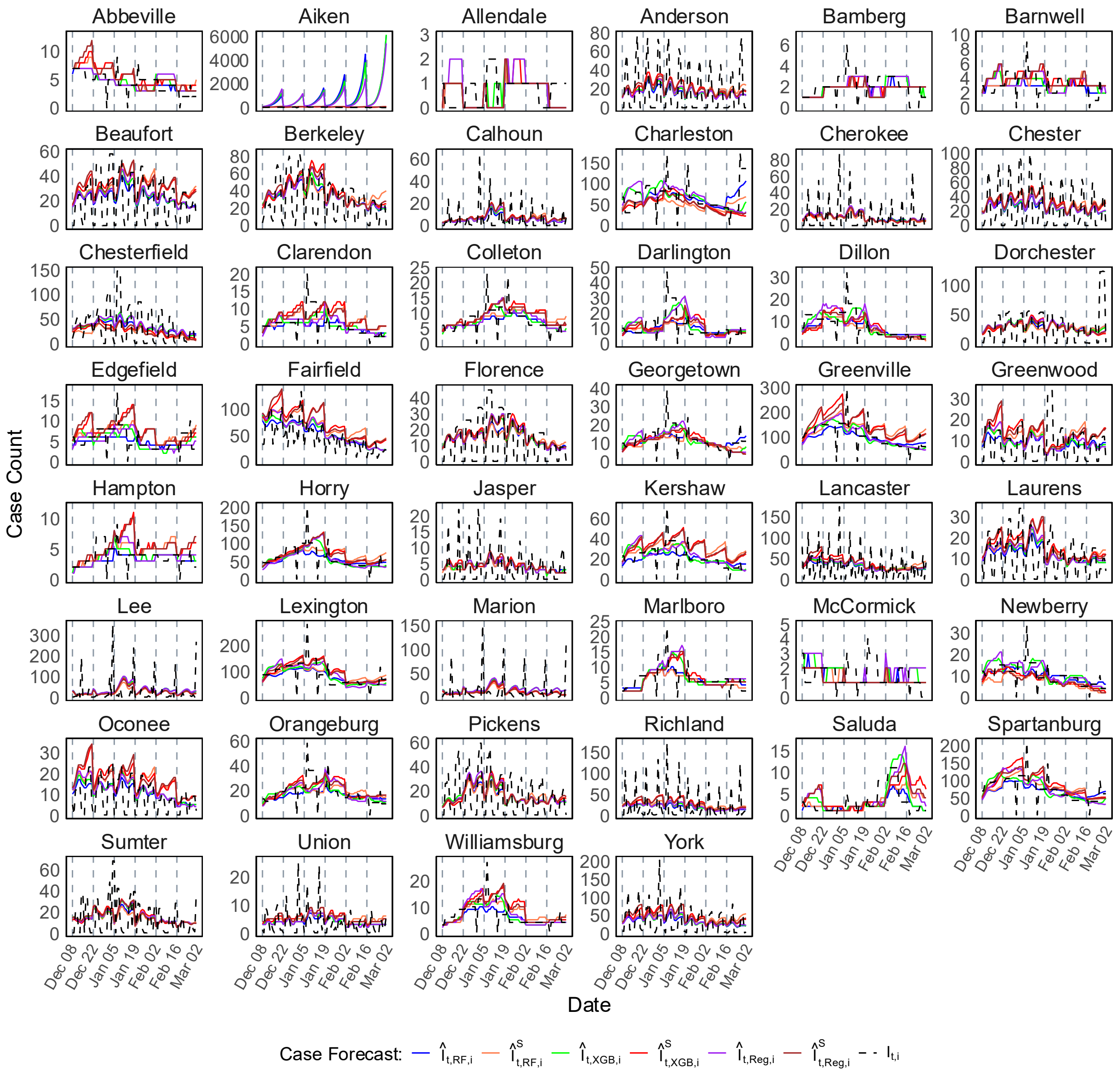

Figure S25. Forecast of COVID-19 case counts at the county level in SC during Scenario-2 (December 11, 2022 – March 04, 2023). This figure presents the forecast of COVID-19 case counts for all 46 counties in SC. The plots compare forecasts generated using multiple individual models against the observed daily case counts (black dashed lines, $I_{t,i}$), where i represents the county and t denotes the time point (day). The forecasts were generated for 14-day ahead predictions over 84 days period using a rolling window approach.

medRxiv preprint doi: https://doi.org/10.1101/2025.06.24.25330210; this version posted June 24, 2025. The copyright holder for this preprint (which was not certified by peer review) is the author/funder, who has granted medRxiv a license to display the preprint in perpetuity.
It is made available under a CC-BY 4.0 International license .

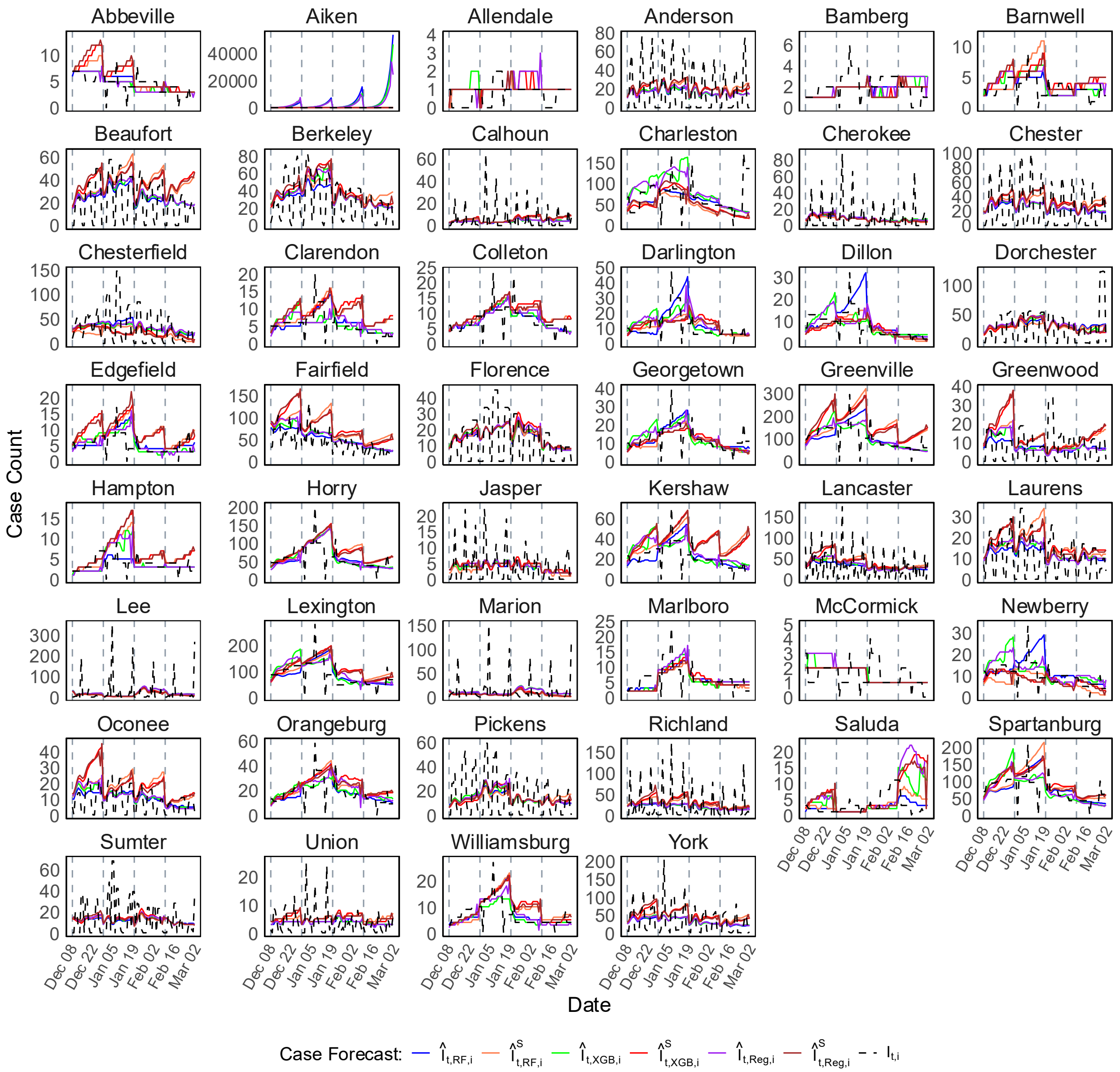

Figure S26. Forecast of COVID-19 case counts at the county level in SC during Scenario-2 (December 11, 2022 – March 04, 2023). This figure presents the forecast of COVID-19 case counts for all 46 counties in SC. The plots compare forecasts generated using multiple individual models against the observed daily case counts (black dashed lines, $I_{t,i}$), where i represents the county and t denotes the time point (day). The forecasts were generated for 21-day ahead predictions over 84 days period using a rolling window approach.
